# Hybrid hemp/glass fiber reinforced high-temperature shape memory photopolymer with mechanical and flame-retardant analysis

**DOI:** 10.1038/s41598-023-44710-6

**Published:** 2023-10-19

**Authors:** Sakil Mahmud, John Konlan, Jenny Deicaza, Guoqiang Li

**Affiliations:** 1 Department of Mechanical and Industrial Engineering, Louisiana State University, Baton Rouge, LA 70803 USA; 2 Department of Mechanical Engineering, Southern University and A&M College, Baton Rouge, LA 70813 USA

**Keywords:** Engineering, Materials science

## Abstract

Cultivated natural fibers have a huge possibility for green and sustainable reinforcement for polymers, but their limited load-bearing ability and flammability prevent them from wide applications in composites. According to the beam theory, normal stress is the maximum at the outermost layers but zero at the mid-plane under bending (with (non)linear strain distribution). Shear stress is the maximum at the mid-plane but manageable for most polymers. Accordingly, a laminated composite made of hybrid fiber-reinforced shape memory photopolymer was developed, incorporating strong synthetic glass fibers over a weak core of natural hemp fibers. Even with a significant proportion of natural hemp fibers, the mechanical properties of the hybrid composites were close to those reinforced solely with glass fibers. The composites exhibited good shape memory properties, with at least 52% shape fixity ratio and 71% shape recovery ratio, and 24 MPa recovery stress. After 40 s burning, a hybrid composite still maintained 83.53% of its load carrying capacity. Therefore, in addition to largely maintaining the load carrying capacity through the hybrid reinforcement design, the use of shape memory photopolymer endowed a couple of new functionalities to the composites: the plastically deformed laminated composite beam can largely return to its original shape due to the shape memory effect of the polymer matrix, and the flame retardancy of the polymer matrix makes the flammable hemp fiber survive the fire hazard. The findings of this study present exciting prospects for utilizing low-strength and flammable natural fibers in multifunctional load-bearing composites that possess both flame retardancy and shape memory properties.

## Introduction

In recent years, there has been growing concern regarding the recyclability of synthetic fibers in fiber-reinforced composites, shedding light on the negative environmental impact of their production^1^. Extensive research has demonstrated the high energy consumption associated with manufacturing carbon fibers and glass fibers, leading to a detrimental carbon footprint^2^. For example, around 400 MJ of energy is required to produce 1 kg of pure carbon fiber^3^. When recycled from carbon fiber-reinforced polymer composites, around 200 MJ/kg of energy is needed, producing a lot of non-disposable residual wastes^2^. In some cases, for instance, recycling glass fibers from thermoset composites is more expensive and requires more energy than producing virgin glass fibers^4, 5^. In this context, using natural fibers as an alternative reinforcement has gained attention, presenting an environmentally friendly solution^6^. By shifting the focus to sustainable alternatives, it can address the challenges posed by plastic waste and work towards a more eco-friendly future. Various options have been proposed to address plastic waste, but they face challenges. (i) Recycling and reusing plastics are difficult due to the presence of diverse polymer types and non-plastic materials^7^. (ii) Producing eco-friendly bio-based plastics from renewable resources is a promising alternative to petroleum-based plastics, but challenges in production and application limit their acceptance^8^. (iii) Combining traditional plastics with bio-based polymers^9^ or plant fibers^10^ has been explored to reduce plastic consumption and wastage. Incorporating renewable natural fibers in plastic products can significantly reduce an equal amount of plastic usage and pollution^11^. As an example, the integration of 20 wt. % natural fibers into plastic items effectively corresponds to a 20 wt. % reduction in plastic utilization. However, natural fibers have limited load-carrying capacity; as a result, our recent report showed around a 30% reduction of tensile strength by adding only 10 wt% cellulose fiber in the polylactide^11^.

The increasing demand for lightweight materials and reduced environmental impact has sparked interest in natural fiber-reinforced polymer (FRP) composites as a feasible substitute for metals and synthetic fiber-reinforced polymers^12^. Glass fibers currently dominate the polymer composites, accounting for approximately 95% of total fiber reinforced polymer composites^13^. However, natural fibers offer numerous advantages, including lower density, biodegradability, wide availability, excellent damping properties, minimal equipment damage, and high health safety, positioning them as an optimistic reinforcing material for polymers. Not only natural fibers but also other plant components, such as leaves^14^ and rice husks^15, 16^, make significant contributions to green composites, serving as cost-effective and eco-friendly alternatives for sustainable product manufacturing. Various life-cycle assessment studies have confirmed the environmental superiority of natural over glass FRP composites^17^. Market projections indicate that the global natural FRP composites market size is expected to reach $10.89 billion by 2024, driven by the growing demand for lightweight and fuel-efficient automobiles^12^. Hemp fibers are such candidates mostly composed of cellulose like other plant fibers. Despite the immense potential of these fibers, they face technical barriers in producing high-performance composites. Challenges include the heterogeneous properties of natural fibers, resulting in variations in the cell wall structure, composition, and geometry, leading to varying fiber quality^18^. Additionally, natural fibers exhibit relatively lower mechanical properties, hydrophilicity that poses compatibility issues and aggregation tendencies in hydrophobic polymer matrices, high water absorption, low thermal stability, and difficulties in processing compared to glass fibers^19^. The hybridization of synthetic fiber could be an excellent idea to overcome the challenges of lower mechanical properties by natural fiber reinforcement^24^.

The properties of polymer composites reinforced with various plant fibers may differ due to variations in inherent fiber characteristics, including tensile strength, moisture absorption, and chemical composition. These distinctions can significantly affect composite properties, leading to variations across materials^25–27^. AL-Oqla et al. recently explored methods for selecting natural fibers in polymeric-based composite materials, including the integrated mechanical-economic-environmental quality of performance and the hierarchy selection framework under uncertainty^20–23^. These reports show that hemp fibers exhibited lower performance than other natural fibers. While hemp fibers exhibit certain technical limitations that impact their performance, such as moisture absorption and compatibility issues with polymers, they offer substantial advantages over traditional glass fibers, positioning them competitively in contemporary industrial applications^28^. Hemp fibers in polymer composites exhibit superior specific stiffness compared to glass fiber composites in both tension and plate bending, with only marginal reductions in specific stiffness compared to carbon fiber composites in plate bending^29^. Bourmaud et al. found that hemp fibers maintain their integrity better than glass fibers during recycling, resulting in minimal loss of mechanical properties in hemp-polypropylene composites^30^.

Fiber hybridization is a method used to improve the sustainability, cost-effectiveness, and performance of natural FRP composites. It involves combining natural fibers with other fibers, either natural or synthetic, that possess superior mechanical, thermal and chemical properties^31^. Hybridization can meaningfully enhance the mechanical performance of natural FRP composites, as demonstrated by improvements in tensile and impact strengths^32^. For example, hybridizing jute fabric with glass fabric at a 1:1 weight ratio resulted in a three-fold increase in tensile strength and a six-fold increase in impact strength over jute fabric-reinforced polyester, while hybridization with carbon fibers yielded nearly ten-fold improvement in tensile strength and five-fold improvement in impact strength^33^. While hybridizing natural fibers with other natural fibers may not yield comparable mechanical improvements, it has value in terms of sustainability. The performance of hybrid composites is influenced by factors such as fiber content, hybrid ratio, fiber orientation, stacking sequences, and innate fiber features.

This study considers fiber orientation and stacking sequences for hybrid reinforcement. According to the previous report^34^, in the case of laminated composite beams subjected to bending load, the top and bottom layers experience the highest normal stress, gradually decreasing towards the mid-plane, where it becomes zero with linear or nonlinear normal strain distribution. While the shear stress is highest at the mid-plane, it is generally not a major concern since polymers can typically withstand such levels of shear stress. For example, Ji et al. investigated the effect of adhesive layer thickness on the energy release rate and traction–separation laws or cohesive laws of adhesively bonded joints under Mode II or shear loading^35–37^. It was found that the shear strength is much higher if the polymer adhesive is in the form of thin film than that in the form of bulk, which is exactly the case when the polymer is used as the matrix in laminated composites. Utilizing this principle, this study developed a hybrid fiber reinforcement approach with a gradient configuration, wherein the top and the bottom surface layers are reinforced with strong glass fiber with a weak natural hemp core as mid-plane. The mechanical properties and chemical composition of both fibers are shown in Table S1. To make the composite multifunctional, this research used a hybridization of natural and synthetic fiber to reinforce the newly synthesized high-temperature shape memory polymer (HTSMP). A UV-curable triacrylate monomer with a thermally stable isocyanurate ring created a highly interconnected network for synthesizing HTSMP^38^. To enhance flame resistance, a small quantity of commercially available phosphine oxide was employed as both a photo-initiator for UV curability and a flame-retardant structure. Unlike traditional phosphorus-based flame retardants like phosphate, phosphine oxide demonstrates exceptional stability against thermal and hydrolysis processes. The resultant HTSMP, featuring a highly crosslinked and uniform network, exhibits a high glass transition, impressive mechanical properties at high temperatures, and remarkable shape recovery stress^38^.

Therefore, the objective of this study is to produce a new multifunctional laminated composite using natural fiber as a counterpart of synthetic fibers with high impact tolerance and shape memory properties. It employs beam theory to effectively integrate weak natural fibers into composites while safeguarding mechanical properties. It also introduces an innovative UV-curing polymer, achieving rapid curing in just 20 s for plant-based polymer composites. Remarkably, the study enhances flame retardancy without resorting to chemical treatments, relying on the specific polymer type and fiber stacking sequence to delay natural fiber burning while preserving structural integrity. This approach contributes to reducing plastic consumption, aligning with sustainability goals for developing high-performance multifunctional photopolymers-based hybrid composite laminates.

## Experimental

### Materials

A mold release agent (fib-release) and the unidirectional glass fibers were purchased from Fiberglast, USA. 100% nature hemp fabric was kindly donated by Ag Center, Louisiana State University, Baton Rouge, USA. The weight of the hemp fabric used was around 200 g/m^2^. Tris[2-(acryloyloxy)ethyl] isocyanurate (TAI; molecular weight, M_w_ = 423.37 g/mol, CAS No.: 40220-08-4) and photo-initiator Diphenyl(2,4,6-trimethylbenzoyl)phosphine oxide (97%) (TPO, M_w_ = 348.4 g/mol), were purchased from Sigma-Aldrich, USA and used as received.

### Fabrication of composite laminates

Initially, a solution for the thermosets was prepared by mixing 93 wt% of TAI monomer and 7 wt% of TPO photo-initiators at 100 °C for 2 h and degassed at 80 °C for 1 h in a vacuum oven. The conditioned solution was then used to wet the glass and hemp fabric of 165 mm × 165 mm dimension. Hemp fibers typically contain approximately 9.1% moisture^39^. Therefore, before the prepreg process of hemp fibers, they underwent oven drying at a temperature of 100 °C for a minimum duration of 4 h to eliminate all moisture. A hand layup technique was employed to produce 5.05 mm thick composites by laying prepreg fabric. Plain glass sheets were pretreated with 15 mL of a mold release agent and dried sufficiently to remove air around them. Two extra open plastic mold frames were placed on both sides to protect the base glass sheets from breakage. Eight C-clamps were employed to apply enough pressure to the laminated composites to keep them in place without shattering the glass sheets. The compacted setup was then delivered to the UV chamber (IntelliRay 600, Uvitron International, USA), where it was cured in just 20 s on each side under 35% irradiation intensity (232 nm, ~ 45 mW/cm^2^) at room temperature. After demolding, the UV-cured laminated composites were thermally post-cured for 1 h at 200 °C under a nitrogen atmosphere. Then, the thermally post-cured composite boards were cut to the required dimensions using a high-precision water-jet cutting machine (WARDJet 5′ × 10′ Waterjet Machining System). The whole procedure is schematically shown in Figure S1: *Supplementary Information*. The FRP composite laminates independently produced by eight layers of natural hemp (H), and six layers of synthetic glass (G) fibers were marked as hFRP and gFRP, respectively. For the hybrid laminates, fabric layers were positioned so the core was natural fibers, and the outer (top and bottom) layers were glass fibers of single and double layers, as remarked them hFRP-G1 (GHHHHHG) and hFRP-G2 (GGHHHGG), respectively. It is worth mentioning that the number of fabric layers was chosen based on the requirement to fill the mold thickness (5.05 mm) while maintaining a constant fiber volume fraction.

### Characterization and measurements

Initially, the composite density was calculated by the density of constituting materials (ρ_i_) and the weight fraction of constituting materials (W_i_) using Eq. (1). Then the fiber and matrix volume fractions were determined using Eq. (2) (Prakash et al.^40^).

1 $${\rho }_{c }=\frac{1}{\sum \frac{{W}_{i}}{{\rho }_{i}}}$$

2 $${V}_{i}={W}_{i}\frac{{\rho }_{c}}{{\rho }_{i}}$$

A Thermo Nicolet Nexus 6700 FTIR spectrometer (Thermo Scientific, USA) was used to record Fourier transform infrared (FTIR) spectra in attenuated total reflection mode, collecting 32 scans at a resolution of 4 cm^–1^ ranging from 650 to 4000 cm^−1^. A PerkinElmer 4000 DSC (MA, USA) was employed to examine the thermal properties to perform a differential scanning calorimeter (DSC) test. Samples weighing 5–10 mg were subjected to a linear heating/cooling rate of 10 °C min^−1^ and a 3-min holding period between each heating or cooling cycle. The heating and cooling process was repeated twice, and nitrogen gas was purged from the system at 30 mL/min. A TGA550 thermal analyzer (TA instruments, DE, USA) was utilized to conduct non-isothermal thermogravimetric analysis (TGA) tests. A sample weighing 5 and 8 mg was heated in a nitrogen environment (25 mL/min) at a rate of 10 °C min^−1^ from 30 to 800 °C.

A low-velocity impact (LVI) test was employed using the DYNATUP 8250HV drop-weight impactor to investigate the impact resistance of the composite samples. The total impactor weight was 6.7 kg. The specimens were sized at 152.40 mm × 25.40 mm × 5.04 mm and were examined at an impact velocity of 2 ms^−1^ according to ASTM D3763-18 standard. The test aimed to measure the energies required for initiating and propagating damage. After that, a compression after impact (CAI) test was conducted to measure the remaining strength after impact. The test was carried out at room temperature in an MTS Q Test 150 machine following a strain-controlled mode with a 1.0 mm/min loading rate. The obtained load–displacement data were translated into stress (σ) -strain (ϵ) values using Eqs. (3) and (4), respectively. These calculations were based on the compressive load (P), cross-sectional area (A), initial length (L_0_) and the change in length (ΔL) of the sample during compression.

3 $$\sigma = \frac{F}{A} = \frac{F}{b \times h}$$

4 $$\varepsilon = \frac{\Delta L}{{L}_{0}}$$

To evaluate the shape memory property, a rectangular FRP composite (114.0 mm × 12.50 mm × 5.04 mm) was placed between the three-point bending test fixture of an MTS Alliance RF/10 machine and heated to 220 °C for 1 h to reach thermal equilibrium. Then, the sample was bent to 2 mm at a speed of 0.5 mm/min and held at that strain for 30 min. The resulting bent sample was quickly cooled to room temperature by spraying water, and the load was removed. This procedure, known as hot programming, was performed above the glass transition temperature (T_g_). The entire hot programming technique is schematically presented in Figure S2. The span length of the bent sample and length after recovery were recorded to compute the shape fixity ratio (F) and shape recovery ratio (R) using Eqs. (5) and (6), respectively^41^.

5 $$F= \frac{{\mathcal{l}}_{0}-{\mathcal{l}}_{2}}{{\mathcal{l}}_{0}- {\mathcal{l}}_{1}}\times 100\%$$

6 $$R= \frac{{\mathcal{l}}_{3}-{\mathcal{l}}_{2}}{{\mathcal{l}}_{0}- {\mathcal{l}}_{2}}\times 100\%$$

herein, ℓ_0_ is the initial span length, ℓ_1_ is the span length after bending, ℓ_2_ is the span length after the load removal, and ℓ_3_ is the span length after shape recovery. In addition to shape recovery test, some of the hot-programmed bent samples were also tested for stress recovery. To conduct a fully constrained stress recovery test on a programmed sample, the MTS fixtures were preheated at 220 °C for an hour to prevent thermal expansion of the test fixtures. The sample was then quickly inserted between the MTS clamps to ensure total confinement and zero recovery strain. The machine then measured the recovery load over a period of time. Flexural stress (σ_f_) at the outer surface at mid-span was calculated during stress recovery test under the three-point bending configuration based on recovery force (P) recorded by the load cell, span length (L), beam width (b), and beam thickness (h) using Eq. (7) (Konlan et al.^42^). Based on the mid-span deflection (δ), Eq. (8) was used to calculate the maximum strain (ε) at the outer surface. Additionally, Eq. (9) was used to calculate the modulus of elasticity (E).

7 $${\sigma }_{f}= \frac{3PL}{{2bh}^{2}}$$

8 $$\varepsilon = \frac{6\delta h}{{L}^{2}}$$

9 $$E= \frac{{PL}^{3}}{4b\delta {d}^{3}}$$

A basic burning test was conducted to assess the flame retardancy of the composite sample. This involved preparing a rectangular shaped sample with dimensions of 76.20 mm × 12.70 mm × 5.05 mm and positioning it horizontally. A gas lighter ignited the sample for 10 s and then 90 s after the first 10-s ignition. The entire combustion process was recorded using a camera. The char residue of the composite sample was further characterized by a scanning electron microscope (SEM, JSL-6610 LV, JEOL USA) and x-ray Photoelectron Spectroscope (XPS, Scienta Omicron ESCA 2SR). The residual bending strength of some samples after 40 s burning tests was also conducted, under the same three-point bending test configuration and test condition.

## Results and discussions

### Composites design and structural characterizations

The fiber volume fraction in FRP polymer composites affects their resultant properties such as strength, stiffness, load transfer efficiency, fracture toughness, thermal expansion, and density^43^. Although higher fiber volume fractions generally result in improved mechanical properties and dimensional stability, it may proceed in insufficient wettability of the reinforcing fibers by the polymer^44^. Depending on the types of polymer and reinforcement, the composite’s fiber volume fractions ranging from 10 to 50% can offer optimal properties. Therefore, the composite laminates were prepared in this study with a total fiber volume fraction of ~ 35%. In hFRP-G1 composites, the laminates consisted of 16.61 vol% of glass fiber and 17.28 vol% of hemp fibers, whereas in hFRP-G2 composites, the laminates included 26.75 vol% of glass fiber and 7.712 vol% of hemp fibers.

Numerous studies have confirmed that polymers demonstrating elevated glass transition temperatures (T_g_) exhibit enhanced dimensional stability and retain their mechanical properties when exposed to high temperatures. T_g_ recorded for the pure polymer, and its FRP composite laminates are given in Table 1. It has been noticed that the T_g_ of the pure polymer was 218.47 °C. However, the single type of fiber reinforcement causes a certain increase in T_g_ (to 224.38 °C and 228.98 °C for hFRP and gFPR, respectively). A similar increase in T_g_ (to 227.37 °C and 224.11 °C for hFRP-G1 and hFRP-G2, respectively) was also observed for the hybrid fiber reinforcement. The reason for increased T_g_ can be explained as the interactions and compatibility between the reinforcing fibers and the polymer matrix which can also restrict polymer chain mobility^45^. For the hybrid composites, which combine different types of fibers, can benefit from synergistic effects. The unique properties of each fiber type complement each other, resulting in an overall increase in T_g_ compared to composites with a single type of fiber. Another reason could be the variable stiffness and thermal conductivity of multiple fibers compared to the polymer matrix^46^. A similar increase in T_g_ have been observed in other hybrid reinforcement^46, 47^.

**Table 1:** Thermal properties of the pure polymer and FRP composites^*α*^.

Samples	T_g_ (°C )	Wt% loss @ 200 °C	T_5%_ (°C )	T_max_ (°C )	Residue wt% @ 800 °C
Pure polymer	218.47	0.001	395.25	421.33	10.07
hFRP	224.38	0.207	313.07	446.29	7.27
hFRP-G1	227.37	0.146	327.57	441.83	21.66
hFRP-G2	224.11	0.118	334.29	441.71	25.93
gFRP	228.98	0.136	393.78	444.25	51.40

^α^T_g_ was recorded from the second heating scan of DSC, and the material’s weight loss as a function of temperature was derived from TGA.

**Figure 1:**
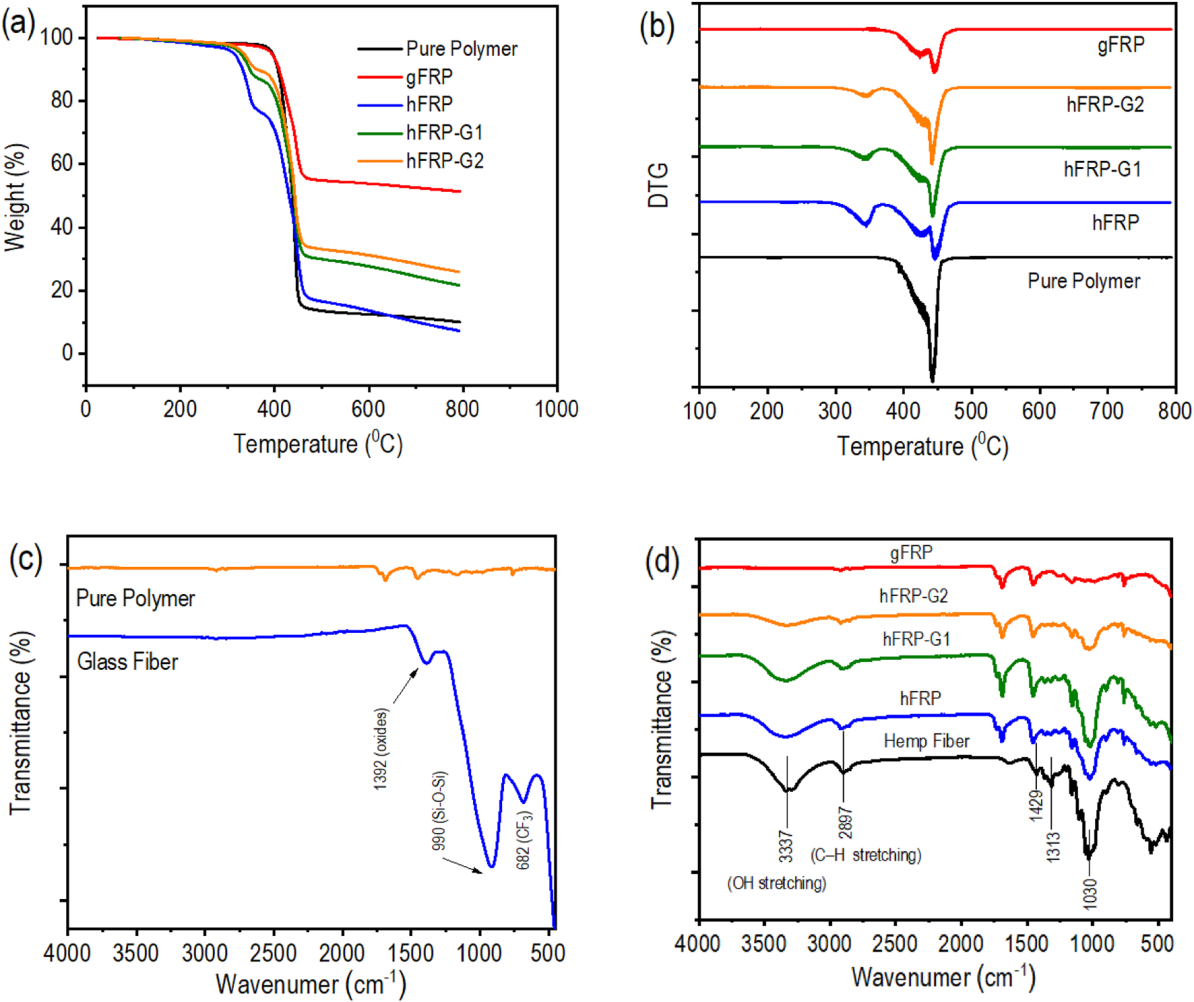
(**a**) TGA curves, (**b**) DTG curves, and (**c**-**d**) FTIR spectra of FRP polymer composite laminates.

As depicted in Fig. 1a, thermogravimetric analysis (TGA) was used to evaluate the thermal degradation of the FRP composite laminates. Figure 1b illustrates the weight loss and the first derivative of the mass ratio (DTG) against temperature. The key parameters, such as the temperature of 5% weight loss (T_5%_) and the temperature of maximum degradation (T_max_), are summarized in Table 1. Accordingly, both the pure polymer and FRP composite laminates demonstrated thermal stability up to 250 °C, even without weight loss at 200 °C. This implies that the maximum curing temperature (200 °C) used in the synthesis and compositions did not significantly impact the materials’ degradation or thermo-oxidative stability. It was observed from T_5%_, the hFRP composite laminates showed comparatively lower temperatures, and it increased with the addition of glass fibers and reached an almost equal value to gFRP. This is because of removing a significant amount of moisture from natural hemp fibers at a temperature ranging from 30 to 250 °C^48^. A similar phenomenon should be observed in T_max,_ but all values become closer due to removing initial moisture. However, all samples follow three stages of degradation. The first stage, which occurs at a temperature up to 250 °C, corresponds to the loss of moisture and dehydration of solvent materials. During this stage, there is a slight weight loss (< 2%) as moisture evaporates from the composite contained natural fibers. The second stage is the samples’ main thermal degradation stage, between 350 and 450 °C. During this stage, the composite material’s polymer matrix and reinforcing fibers begin to decompose and release volatile byproducts. At these temperatures, the decrease in mass could be attributed to the disintegration of an organic sizing additive present in the fiber. In the case of samples reinforced with natural hemp fiber, the decline was caused by the breakdown of hemicellulose, cellulose, and lignin of the plant fibers. The third and final stage begins at 450 °C temperature and onwards is characterized by a plateau in the TGA curve. This plateau represents the residual mass of the composite material that remains after all of the polymer and reinforcing natural fibers have decomposed. The residual mass is typically composed of inorganic fillers, additives, or other non-volatile materials in the composite. As a result, the sample containing more glass fibers (inorganic fillers) produced more residue. In addition, Table 1 provides information on the char yield of the composites, which increases as the quantity of glass fiber increases. This rise in char yield is directly linked to flame retardancy, meaning that a greater amount of char can impede the generation of combustible gases, lower the exothermic nature of the pyrolysis reaction, and inhibit the thermal conductivity of burning objects^49^. The details of flame retardancy performance will be explained in a separate section.

Fourier Transform Infrared (FTIR) spectra were analyzed to study the chemical composition of materials and the interaction between fibers and polymer. As shown in Fig. 1c, the pure polymer does not contain any peak around 810 cm^−1^ of C=C groups, implying the polymerization of C=C groups of TAI monomers. The absence of a peak of the C=C bonds suggests the polymer was successfully synthesized with full curation^38^. The pure glass fibers exhibited various oxides (at 1392 cm^−1^), such as boron oxide and aluminum oxide. The spectra also displayed the presence of an oxygen-silicon bond in the Si–O–Si group at a frequency of 990 cm^−1^ and an asymmetric deformation vibration of the –CF_3_ group at a frequency of 682 cm^−1^ ^50^. Moreover, pure hemp fiber showed a basic cellulose structure, characterized by a wide peak in 3337 cm^−1^ caused by the hydroxyl group and the bound O–H stretching vibration in both the hemicellulose and cellulose ingredients (Fig. 1d). Other significant peaks at 2897 cm^−1^ (C–H symmetrical stretching), 1429 cm^−1^ (HCH and OCH in-plane bending vibration), 1313 cm^−1^ (CH_2_ rocking vibration), and 1030 cm^−1^ (C–C, C–OH, C–H ring and side group vibrations) were also noticed^51^. On the other hand, composite prepared with glass fibers showed spectra similar to those of pure polymers (Fig. 1d). No spectral shift, appearance of new peaks, or disappearance of existing peaks happened due to the addition of glass fibers. This indicates that there were no chemical reactions between the glass fibers and the polymer matrix and that the glass fibers were only physically embedded. This observation has also been reported in previous studies^10, 49^. Similarly, the addition of hemp fibers causes a specific appearance of a peak in the region of 3337 cm^−1^ due to the O–H stretching vibration from cellulose. The peak intensities were increased with the increase in cellulose content. Because this fingerprint is from the hemp fiber, it is believed that, like glass fiber, there is no chemical reaction between the hemp fiber and the polymer matrix.

### Mechanical properties

**Figure 2:**
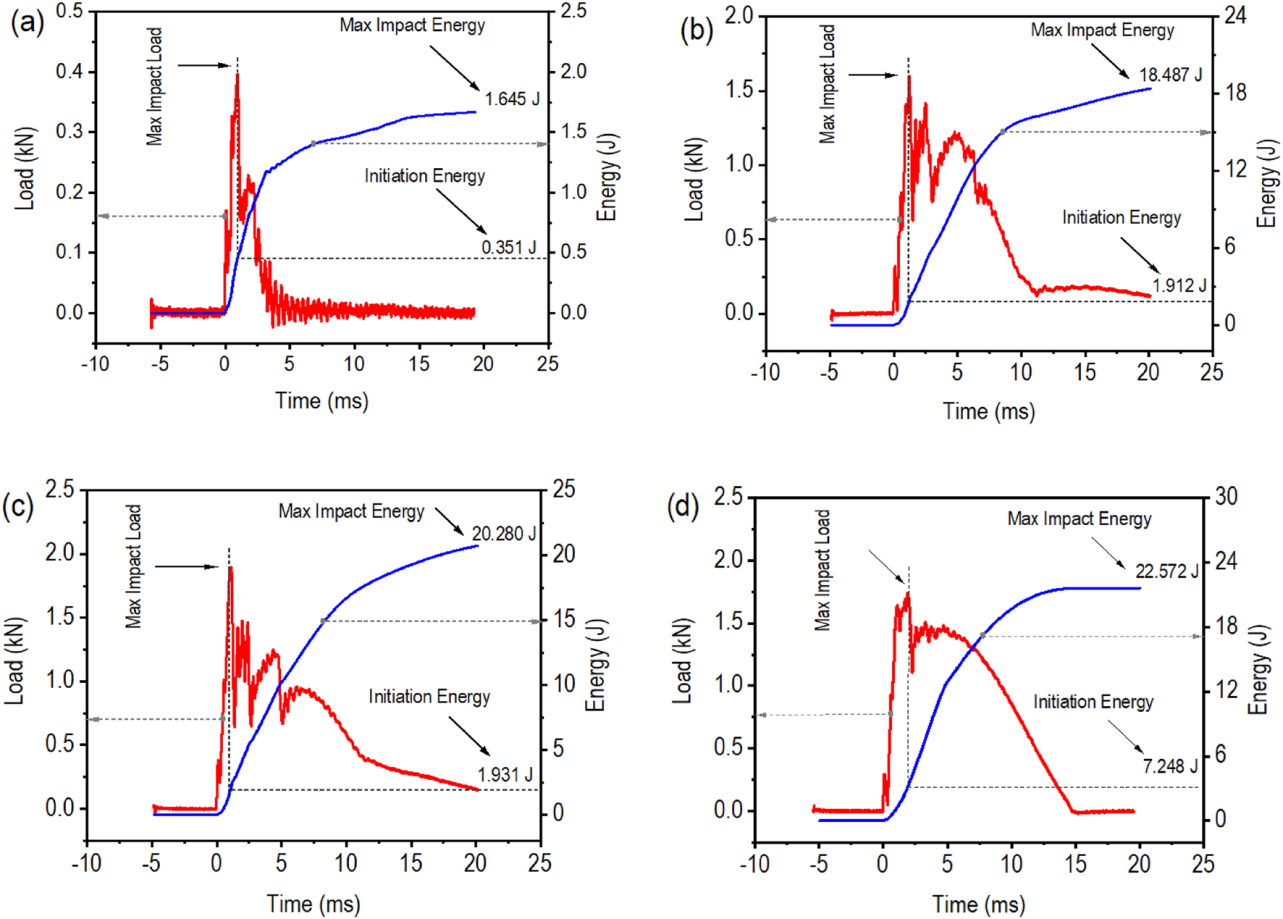
The load and energy traces of LVI: (**a**) hFRP, (**b**) hFRP-G1, (**c**) hFRP-G2, and (**d**) gFRP laminate composites.

The load versus time plots for all FRP composite laminates impacted up to penetration demonstrated apparently similar patterns with at least two major peaks (Fig. 2). According to numerous reports^52, 53^, the abrupt reduction in load (known as the incipient damage point) depicted in these curves signifies the occurrence of damage characterized by the initiation of delamination and an abrupt decrease in stiffness. This threshold load increases with the addition of glass fibers in hFRPs laminates. Following this incipient failure, the curve grew up with fluctuations and eventually reached a peak value. This highest load signifies the maximum amount of force (maximum impact load) that the sample can withstand before undergoing significant fracture, and it was observed to rise significantly as the number of glass fiber layers was added to the hFRPs laminates.

Based on the typical load versus time and energy versus time responses (Fig. 2), important findings such as maximum impact force, initiation energy, maximum impact energy, and propagation energy were presented in Table 2. The impact results can be applied to assess the tolerance and ability of composite laminates to withstand fracture by extrapolating initiation and propagation energies. The initiation energy can be defined as impact energy at the point of maximum force, while the propagation energy is the difference between the initiation energy and the maximum impact energy. Previous research suggests that initiation energy measures the target’s elastic energy transfer capacity, while propagation energy reflects the energy absorbed by the target to cause and spread significant damage^54^. Generally, higher propagation energy values indicate more extensive damage^55^. Based on the above-mentioned theory, the following findings can be sorted from Fig. 2. First, hFRP composite laminates exhibit lower impact tolerance than gFRP composite laminates, as evidenced by the considerably lower maximum impact force and initiation energy of hFRP composite laminates. Moreover, the increased propagation energy of gFRP composite laminates implies that they absorb more energy through damage. Secondly, the inclusion of glass fiber layers in hFRP composite laminates directly raises the maximum impact force and initiation energy, signifying an associated improvement in impact tolerance. Additionally, the increased propagation energy resulting from the integration of glass fiber and hemp fiber layers suggests that damage in these hybrid composites is primarily caused by delamination.

**Table 2:** Mechanical properties of FRP composite laminates based on LVI test.

Samples	Maximum impact load (kN)	Initiation energy (J)	Max impact energy (J)	Propagation energy (J)	Ductility index (DI)
hFRP	0.405 ± 0.012	0.351 ± 0.081	1.645 ± 0.170	1.294 ± 0.089	3.68
hFRP-G1	1.742 ± 0.092	1.912 ± 0.119	18.487 ± 3.351	16.575 ± 3.231	8.67
hFRP-G2	1.963 ± 0.096	1.931 ± 0.218	20.280 ± 0.584	18.349 ± 0.365	9.50
gFRP	1.750 ± 0.249	7.248 ± 1.934	22.572 ± 0.803	15.325 ± 1.130	2.11

^α^A minimum of five valid trials were conducted for each sample group to compute the mean and standard deviation (±).

The ductility index (DI) of most materials is typically calculated as the ratio of energy absorbed after reaching the maximum load to the energy absorbed up to the maximum load. However, as seen in Fig. 2, the failure process of laminate composites began earlier than the maximum load. Thus, it may be more logical to consider the energy at the yield point, denoted as initiation energy (E_i_), and the energy dissipated after the yield point, denoted as propagation energy (E_p_)^56^. So, the DI = E_p_/E_i_, indicates the material’s ductility. In FRP composites laminates, the reinforcement fibers, i.e., hemp and glass fibers, provide the ultimate strength and stiffness, while the polymer matrix provides protection and transfers stress between the fibers. The DI reflects the combined behavior of the fibers and the polymer matrix. As given in Table 2, hFRP composite laminates showed a DI of 3.68, which was increased up to three folds upon stacking glass fibers layers on its outer surface. The increased DI indicates that the composite laminate can undergo greater plastic deformation before fracture^57^. This is desirable in many engineering applications because it allows the material to absorb more energy and provides a warning sign or gradual failure mode rather than a sudden, catastrophic failure.

The failure patterns of composite laminates due to the impact load application are presented in Figure S3. The damage mechanism in traditional FRP composite laminates is highly intricate. Regarding composite laminates, impact-induced damage typically originates on the non-impacted side or as internal damage. The characteristics of individual components and the interfaces between the fibers and matrix can influence the threshold energy or stress needed to initiate different types of damage resulting from impacts^58^. The primary damage modes of the FRP composite laminates are delamination and matrix cracks. In the case of hFRP, the energy absorption mechanism differs from delamination or matrix cracking and instead relies on fiber fracture (Figure S3-a). When subjected to impact forces, the hFRP composite laminates fracture into multiple pieces due to the comparatively weak strength of natural fibers, a small fiber aspect ratio, and the bidirectional twisted distribution of hemp fibers within the yarn or fabric. This leads to inadequate stress transfer from the matrix to the fibers, resulting in a lower maximum impact load than other specimens (Table 2). In the hFRP-G1 composite laminates (Figure S3-b), both shear cracks (in the weak core of natural fibers) and wide-opened delamination (especially between the interface of natural and synthetic fibers) were induced during impact. On the other hand, the double layers of glass fibers over the natural fiber core (hFRP-G2) can prevent mid-plane shear cracks of the matrix, but delamination was still induced upon impact (Figure S3-c). This wide-opened delamination signifies the importance of a capitalizer for robust interfacial adhesion between natural and synthetic fibers. Delamination in composite structures typically arises from a combination of factors, including matrix cracks, interlayer shear stress at the interface, stiffness mismatch between adjacent layers, the grouping of layers, and laminate deformation^58^. These factors collectively result in incompatible bending stiffness between neighboring layers, especially when they possess varying orientations of reinforcing fibers. Hence, the above-mentioned wide-opened delamination between the interface of bidirectional natural fibers and unidirectional synthetic glass fibers can be minimized by using all unidirectional fibers in the hybrid composite laminates. The concept was simply reflected in unidirectional gFRP composite laminates, which induced negligible delamination upon impact (Figure S3-d).

After the low velocity impact, the delaminated sub-laminates were observed by an optical microscope; see Figure S4. It is seen from Figure S4 (a) that the hybrid composite has a morphology with continuous glass fiber reinforcement, which is typical for continuous fiber reinforced polymer composites. In Figure S4 (b), a smooth fracture surface on the glass fiber reinforced sublaminate is seen due to delamination at the glass fiber/hemp fiber interface. This suggests that the polymer may not have a very strong interfacial bonding with the glass fibers. Further enhancement in the interfacial bonding strength between the glass fiber and the polymer matrix would further increase the load carrying capacity of the damaged composites. In Figure S4 (c), it is seen that the fracture surface on the hemp fiber reinforced sublaminate is rough, even some glass fibers can be seen on this side of delamination, suggesting that the hemp fiber has good interfacial bonding strength with the polymer matrix. This can be further validated by Figure S4 (d), which shows that the polymer has penetrated into the space between the twisted hemp fibers.

**Figure 3:**
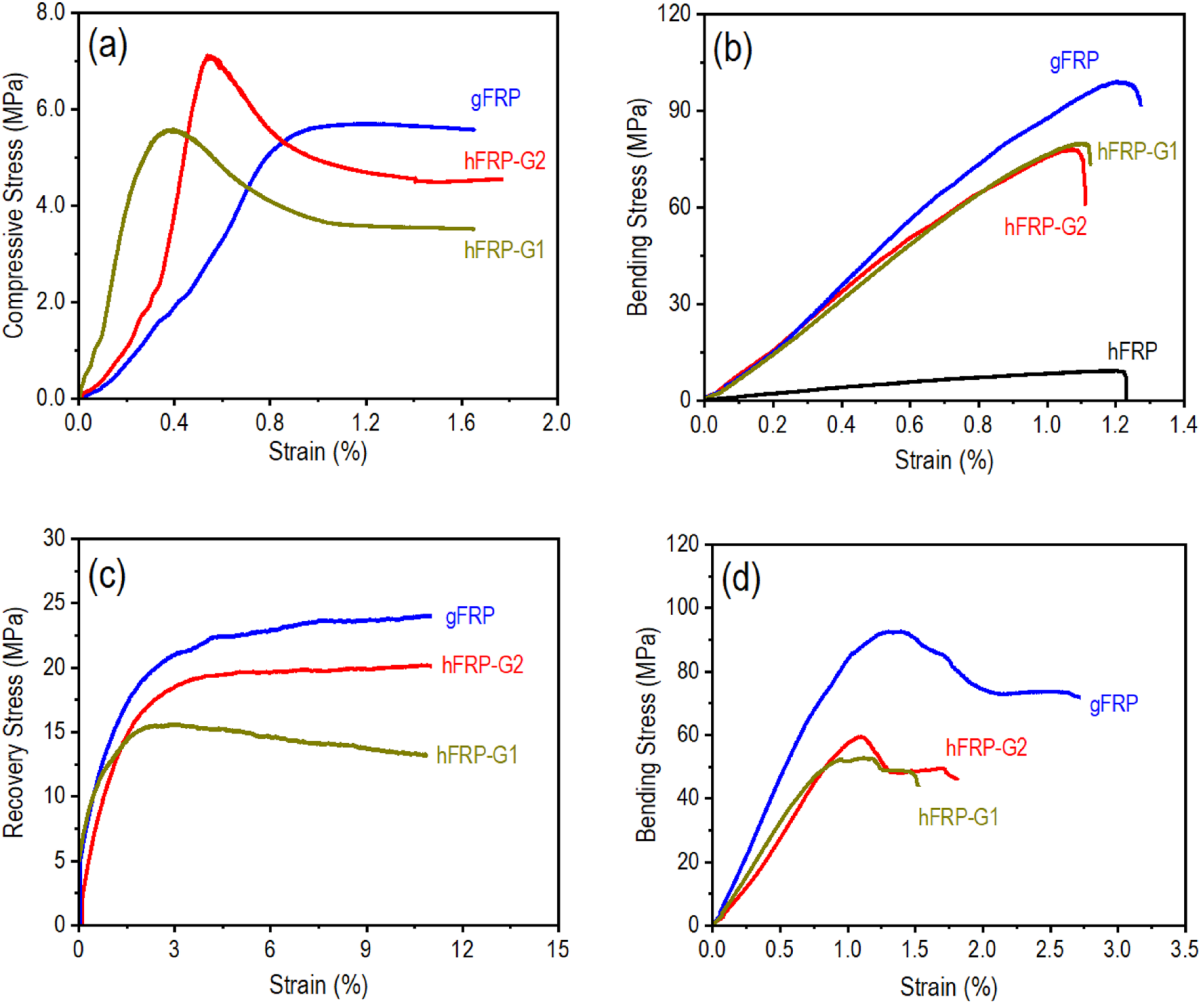
(**a**) Compressive stress versus strain curve from the compression after impact test, (**b**) bending stress versus strain curve from the three-point bending test during hot-programming, (**c**) recovery load versus time of laminate composites after programming, and (**d**) bending stress versus strain curve from the three-point bending test on burned samples.

The compression after impact (CAI) was performed to assess the strength that remains after an impact and the effectiveness of recovery in a confined shape. Earlier research revealed a significant decrease in the compressive strength of materials following LVI compared to undamaged samples^59^. The strength that remains in the sample after the impact is residual strength. The hFPR composite laminates were not considered for CAI since they were broken into several pieces in an LVI test. As presented in Fig. 3a, all samples but gFRP composite laminates show three distinct regions on the CAI curve. In region-I, the applied compressive load was relatively low, and all composite laminates behaved elastically. It means all composite laminates deform under compression but return to their original shape once the load is removed. The curve shows a nearly linear response, indicating a high degree of elasticity. In region-II, especially for the hybrid composite laminates (hFRP-G1 and hFRP-G2), as the compressive load increases, the laminate starts to experience permanent deformation and damage due to the impact event. A reduction in strength and stiffness occurs in this region as the laminate’s weak natural fiber core undergoes progressive damage. As a result, the curve shows a downward trend as the damage accumulates, reflecting a decrease in the hybrid composite laminate’s load-carrying capacity. Since gFRP composite laminates were prepared by single types of fibers (glass) and there was no weak natural fiber (hemp) in the core, this region was merged into the next region. In region-III, the compressive load of samples reaches a relatively constant value. It indicates that the damage propagation has stabilized, and the remaining strength of the composite laminate has reached a relatively stable level. The curve shows a slightly declining trend as compression continues, indicating that the laminate has reached its maximum damage extent and can sustain the applied compressive load without a further significant reduction in strength. Previously a similar pattern of the CAI curve with three distinct regions was also reported while other reinforcements were used in polymer composites^59^. However, the buckling load is a crucial parameter that characterizes the behavior where FRP composite laminates may reach a critical point, and their structural integrity is compromised, leading to buckling. It was observed that the hybrid composite laminates with double layers of glass fibers (hFRP-G2) have an increased buckling load (~ 900 N), and even the single layers of glass fibers in hybrid composite laminates (hFRP-G1) can maintain an almost similar level of buckling load as the gFRP composite laminates (~ 700 N). This demonstrates the positive effect of hybrid and gradient arrangement of natural and synthetic fibers in composite laminates on enhancing the impact resistance.

While the three-point bending was employed for shape memory hot programming, the composite laminate showed a favorable ability to deform under this specific mechanical condition. Based on the typical stress–strain curves (Fig. 3b), critical mechanical properties such as maximum bending load and stress are summarized in Table 2. Generally, various factors, including ply stacking sequence, fiber orientation, fiber volume fraction, and porosity, can influence the mechanical properties of FRP composite laminates. This study investigated the mechanical properties by subjecting the panels to a three-point bending test, focusing on the types of fibers used and their stacking sequence. The maximum bending stress of the composite laminates varied depending on the fibers employed. Neat glass fabric (gFRP) exhibited a higher bending stress (∼86.28 MPa) due to the superior strength of glass fibers. Conversely, pure hemp fibers (hFRP) showed the lowest bending stress (∼10.49 MPa), owing to the lower strength of natural fibers. Hybridized FRP composite laminates hFRP-G1 and hFRP-G2 demonstrated around seven–eight folds higher bending stress (∼ 75 MPa) than hFRP. While composite laminates made of natural fiber alone (hFRP) significantly suffer from the low load-bearing capacity, only a single layer of glass fibers stacking over the natural fiber core (hFRP-G1) significantly increases the bending load. It even exceeds the composite laminates made of the strongest glass fiber alone (gFRP) when double layers of glass fibers are stacked over the natural fiber core (hFRP-G2). In addition, the modulus of elasticity (E) calculated using Eq. (9) was 0.72, 66.78, 52.85, and 52.94 GPa for hFRP, hFRP-G1, hFRP-G2, and gFRP, respectively. The LVI test described above also observed a similar pattern of mechanical properties. The outer layers in the hybrid composite, reinforced with the strongest glass fabrics, effectively carried the maximum stress during the three-point bending. As a result, the glass fibers effectively resisted both compressive and tensile stress. On the other hand, the neat hemp fiber-reinforced (hFRP) composite exhibited the lowest bending load and stress values due to the low strength of natural fibers, and their bidirectional arrangement in the fabric, resulting in the lowest load-carrying ability. In summary, the stacking sequence of fiber layers in the composite laminates allowed for an effective bending stress distribution. The top and bottom layers, reinforced with the strongest glass fibers, experienced the highest normal stress. In contrast, the layers in the midplane, containing the weak natural hemp fibers, experienced zero normal stress. The shear stress was highest at the midplane under the three-point bending load, but its significance was diminished as most polymers can withstand higher shear stress, particularly when the polymer layer thickness is thin. Using hFRP-G2 as an example, the maximum shear stress in the mid-plane can be calculated by τ_max_ = (3 V/2bh), where V is the transverse shear load, b is the width and h is the height of the laminated composite beam. In hFRP-G2, V = (363.44 N/2), b = 12.07 mm, and h = 5.04 mm, hence, τ_max_ = 4.48 MPa, which is a small number as compared to the shear strength of thermoset polymers. Based on Feng and Li^38^, the tensile strength of this polymer after 40 s UV curing is 32 MPa. Based on Tresca theory, the shear strength of the polymer is 32/2 = 16 MPa, which is much higher than the shear stress (4.48 MPa) in the mid-plane of the hFRP-G2 laminated composite. Although the polymer matrix has a high shear stretch, the interfacial bonding strength between the polymer and the fibers is low. As discussed in the FTIR results, there is no chemical bonding between the fibers and the polymer. Therefore, under low velocity impact, delamination starts from fiber/matrix interfacial debonding. Figure S5 is the surface of the delaminated glass fiber reinforced sub-laminate in the hFRP-G2 composite. It is seen that the polymer matrix debonded from the glass fibers. Therefore, increasing the interfacial bonding strength between the polymer and the fibers, both glass fiber and hemp fiber, can further increase the bending strength of the composites.

### Shape memory effect

**Figure 4:**
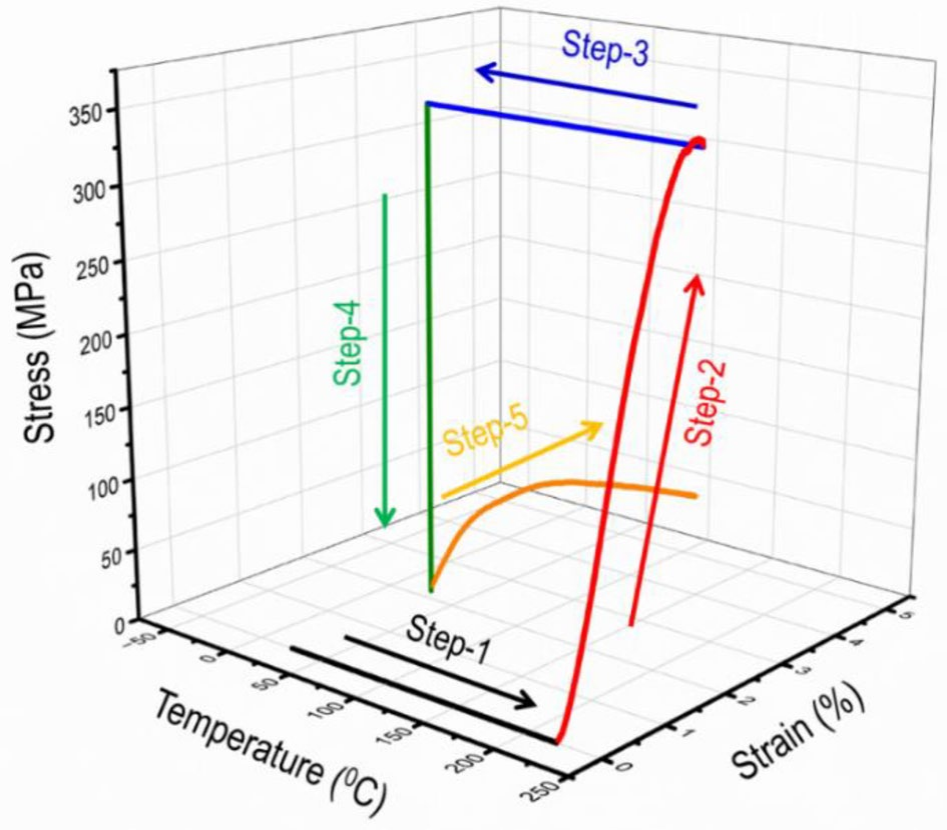
Typical five-step thermomechanical cycles for the gFRP composite laminates. Step 1: Heating from room temperature to 220 °C. Step 2: high-temperature loading. Step 3: Cooling from 220 °C to room temperature. Step 4: Unloading. Step 5: Fully constrained stress recovery.

The entire shape-memory programming and constrained stress recovery cycle are presented in Fig. 4. For shape memory polymers, the polymers will not exhibit shape memory effect without programming, which is a process of mechanically deforming the polymers at a certain temperature. For thermoset shape memory polymers like the one used in this study, they can be programmed at temperature below the glass transition, called cold programming, or within the glass transition zone, called warm programming, or above the glass transition zone, called hot programming^60, 61^. In this study, hot programming was used. In hot programming, it consists of the four steps as shown in Fig. 4, including Step 1: heat the sample to 220 °C, which is above the glass transition zone; Step 2: at the hot temperature, load the sample to the designed bending strain of about 3% and hold the strain for about 30 min to allow for structural relaxation; Step 3: cool down the sample to room temperature, which is a temperature below the glass transition zone, while holding the bending stress constant; Step 4: remove the load by unloading. These four steps completed the hot programming process. In Step 5, it can be either free shape recovery, which allows shape recovery without any constraint, or fully constrained shape recovery or stress recovery, which does not allow any recovery strain to occur, or partially constrained shape recovery, which allows some strain to recovery, and releases some recovery stress. In Fig. 4, Step 5 is a fully constrained shape recovery test. The sample was re-heated to 220 °C while not allowing any strain to recovery, leading to a recovery bending stress of about 24 MPa. The five steps are called a thermomechanical cycle. To evaluate the shape memory behavior, the pre-programmed bent samples were placed in the three-point bending fixture and heated to 220 °C. Then the recovery force with respect to time was recorded by the load cell. The stress recovery curve illustrates the recovery process of the deformed FRP composite (Fig. 3c). When the oven temperature reaches 220 °C, it triggers the recovery of the pre-programmed memory; as a result, the sample starts to recover. Because of the constraint by the fixture, free shape recovery is not allowed. Hence, the recovery load gradually increases over time. All curves typically show an initial rapid increase in recovery load, followed by a more gradual increase until it reaches a plateau. The plateau indicates that the material has reached its maximum recovery and can no longer regain its original shape beyond that point. The recovery stress was calculated by the same bending stress formula of the beam (Table 3). It was observed that the recovery stress achieved by the single glass fiber layer stacked over the nature hemp core (hFRP-G1) was lower (~ 16.65 MPa) than the composite laminate made by glass fiber alone (~ 21.39 MPa). However, this value increases to ~ 24.17 MPa when double glass fibers layers are stacked over the nature hemp core (hFRP-G2), and these results are consistent with the maximum bending stress determined during three-point bending test. A similar level of recovery stress has been reported previously for the other shape memory polymer composites^62–65^. It was also reported that the recovery stresses displayed a wide range, depending on whether they belonged to pure polymers or FRP composites^66^. This offers researchers and engineers a diverse selection of pure shape memory polymers (SMP) or composites catering to their specific design requirements.

**Table 3:** Mechanical properties based on three-point bending tests of virgin specimens and specimens after burning^δ^.

Samples	Max bending stress (MPa)^α^	Max recovery stress (MPa)^α^	Max bending stress (MPa)^β^	Residual bending stress (%)^γ^
hFRP	10.49 ± 1.16	–	–	–
hFRP-G1	75.92 ± 8.26	16.65 ± 2.19	50.70 ± 4.36	66.78
hFRP-G2	73.63 ± 7.11	24.17 ± 1.43	61.50 ± 12.09	83.53
gFRP	94.25 ± 5.89	21.39 ± 4.11	90.57 ± 0.44	96.10

^α^Results from the three-point bending test on the virgin sample, ^β^ Results from the three-point bending test on the burnt sample, and ^γ^The residual bending stress test on the burnt sample compared to the virgin sample. – Undetected value due to the sample breakdown in the previous step. ^δ^A minimum of five valid trials were taken into account for each sample group to compute the mean and standard deviation (±).

In the numerical estimation, the shape fixity ratios of the gFRP, hFRP-G1 and hFRP-G2 are found to be 52.80%, 58.28%, and 53.99%, and the shape recovery ratios are 71.58%, 86.02%, and 70.28%, respectively. However, the pure polymer demonstrates a similar level of shape fixity ratio (~ 58.0%), but in terms of shape recovery ratio, it shows a higher percentage (~ 93.1%) compared to the polymer composite^38^. A similar phenomenon was observed in metallic open-cell foam composites with another SMP^67^. The reason is that the fiber itself has no shape memory effect, therefore, it resists the recovery of the SMP matrix, leading to lower shape recovery ratio than that in the pure SMP matrix.

### Flame retardancy

**Figure 5:**
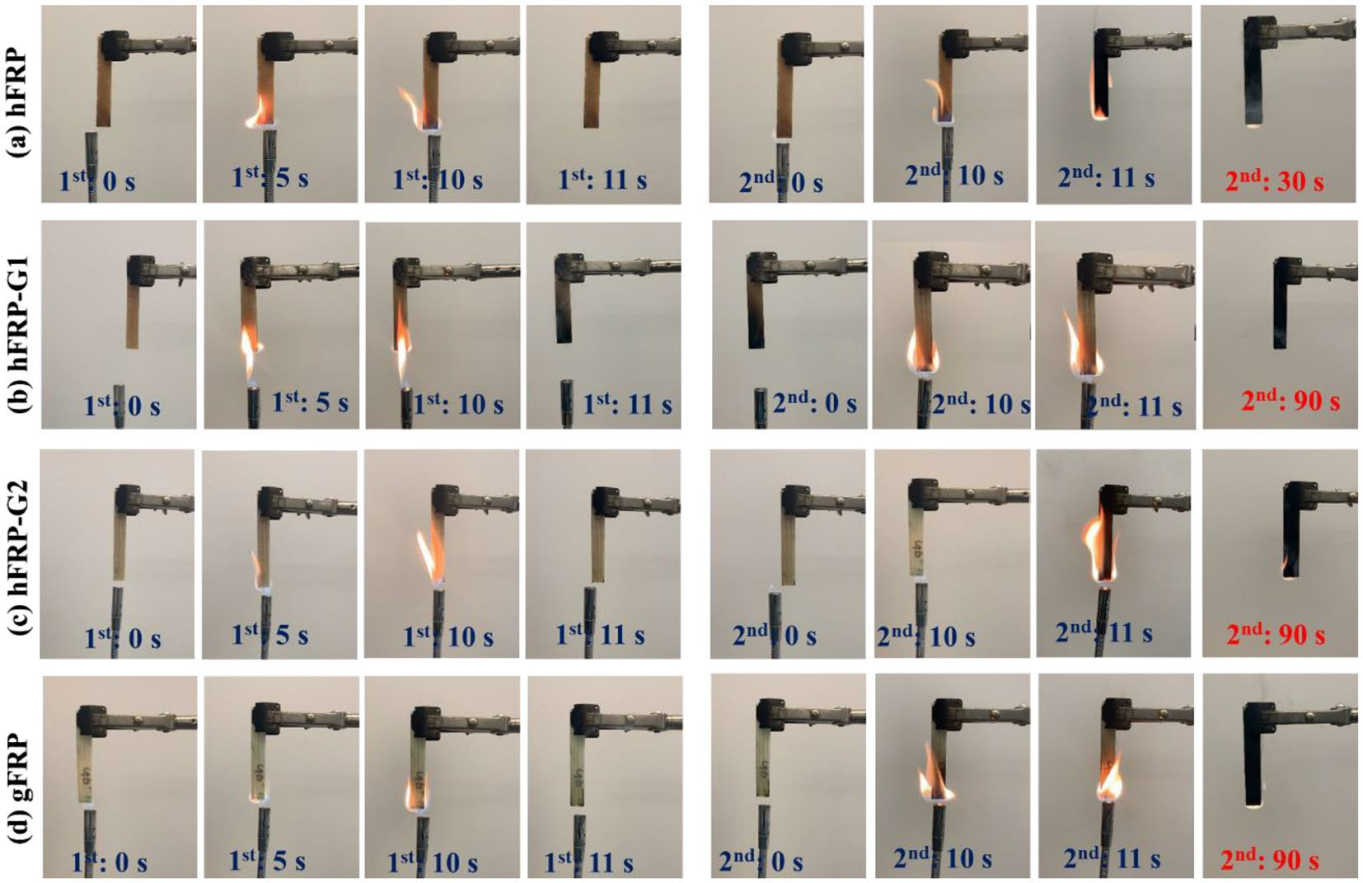
Vertical ignition test of (**a**) hFRP, (**b**) hFRP-G1, (**c**) hFRP-G2, and (**d**) gFRP composite laminates.

In this section, the flame retardancy test results are detailed analyzed. FPR composite laminates were subjected to a flame retardancy test through vertical ignition and burning (Fig. 5). The flame retardancy of gFRP composites is generally considered good due to the inorganic nature and high char formation efficiency of glass fibers^68^. As a result, the sample made of entirely glass fibers (gFRP) cannot be ignited in the first 10 s ignition process. In the case of hybrid laminates, natural fibers were protected by glass fiber layers, so it can also assume that glass fibers delayed the burning of natural fiber core; consequently, they also cannot be ignited in the first 10 s ignition process. The flame retardancy of natural FRP polymer composites is lower compared to synthetic FRP polymer composites. This is due to the various constituents present in natural fiber such as cellulose, hemicellulose, and lignin^69^. However, throughout the initial 10-s ignition phase, it is noteworthy that the hFRP composite laminates also maintained their non-combustible state. The ignition and combustion of composite materials are contingent upon both the composition of the reinforcement and the type of polymer used. Herein TAI monomer and TPO photo-initiator has been used to synthesize the polymer. The flame retardancy of the hFRP composites can be ascribed to the thermally stable isocyanurate rings present in the TAI monomer and the significant phosphorus content in the TPO molecule^38^. Moreover, during the second ignition, the gFRP and hybrid composite laminates cannot be ignited in the first 10 s, and the flame is instantly extinguished after removing the lighter. When a continuous flame supply continued for 60 s, these samples were ignited, and the flame could retain for another 30 s. In the case of hybrid composite laminates, the flame can propagate through the mid-plane (natural fiber) and last for 30 s. On the other hand, during the second burning phase, the hFRP composite laminates can ignite in 10 s and burn out in 30 s after removing the lighter. In summary, the findings indicate that the shape memory polymer matrix exhibits exceptional thermal stability and resistance to thermal oxidation, primarily due to a thermally stable triazine ring and aromatic structures. These characteristics imply that the sample is highly resistant to ignition and possesses excellent flame-retardant properties.

**Figure 6:**
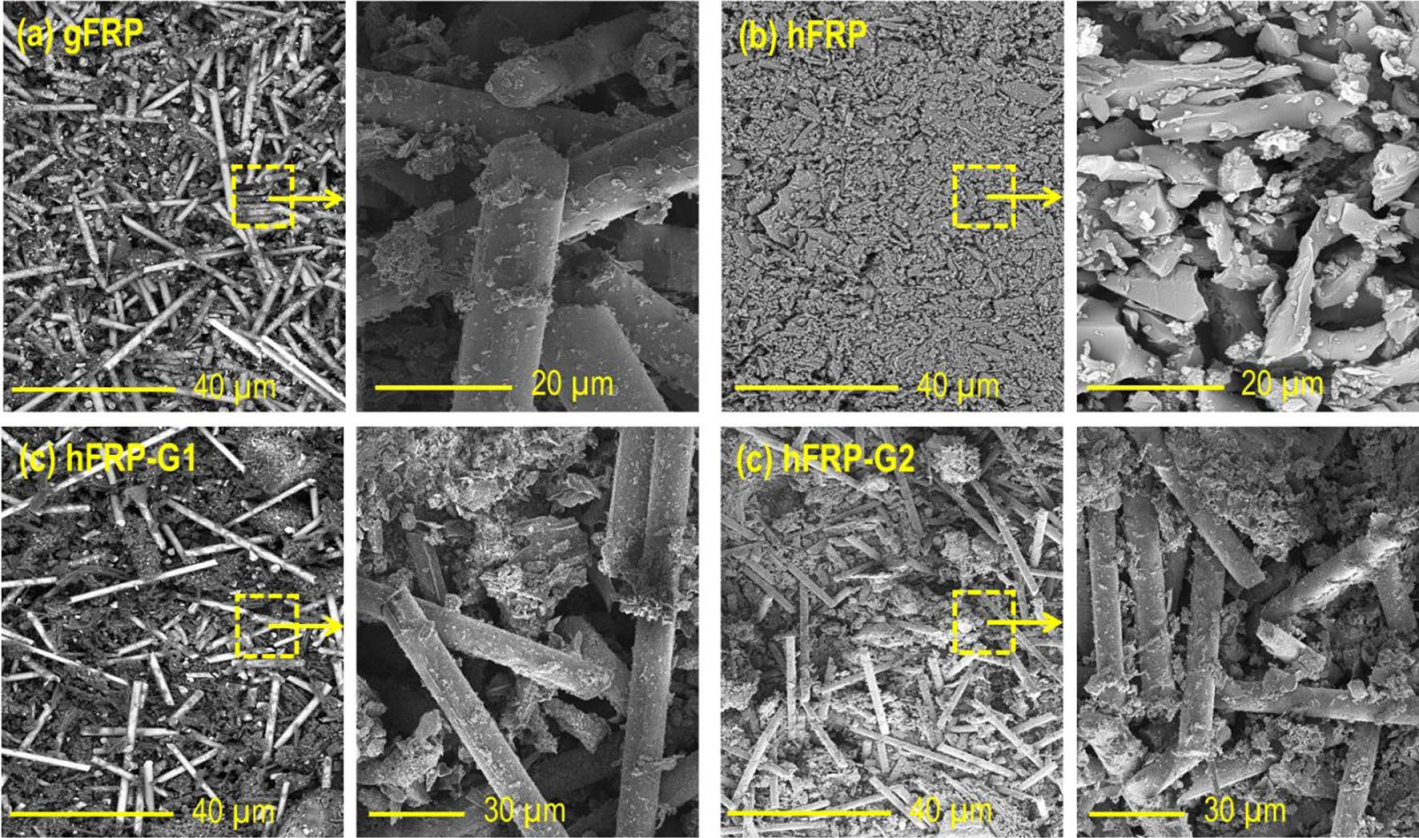
SEM images of char residues of (**a**) gFRP, (**b**) hFRP, (**c**) hFRP-G1, and (**c**) hFRP-G2 laminate composites.

Morphological analysis using SEM on the char residues of the laminated composites have been conducted to examine the mechanism behind flame retardancy and the structure of the resulting char (Fig. 6). The SEM images of the char residue from the gFRP appear undamaged and continuous. However, this image reveals that a significant portion of the laminates’ combustion involved the polymer matrix. The glass fiber within the char was predominantly intact and smooth, with some polymer char residues. On the other hand, the char residue from the hFRP shows complete char contributed by polymer and natural fibers. When natural fibers are exposed to flames, cellulose undergoes thermal degradation, breaking into volatile and combustible gases. This decomposition process weakens the fiber structure and hampers the formation of a cohesive and stable char layer. However, the char of hybrid composite laminates (hFRP-G1 and hFRP-G2) contained a proportional portion of intact glass fibers and char residues contributed by natural fibers and polymer. This confirms that the fire-resistant and inorganic nature of the glass fibers influenced this type of combustion, thereby enhancing the composite’s flame retardancy compared to the pure polymer. In conclusion, glass fiber resists flame through its inorganic composition, high melting point, thermal stability, and ability to help form a protective char layer, making them resistant to heat, combustion, and fire propagation. Their non-combustible nature and limited smoke and toxic gas release further enhance their fire safety characteristics.

The flame retardancy properties of the composite laminates are not only associated with the inorganic glass fibers but also with the polymer used in the matrix. Our previous research^38^ demonstrated consistent results in TGA tests for synthesized high temperature shape memory polymer (HTSMP), regardless of whether the tests were conducted in air or an argon environment. The polymer exhibited stability even under attacks from thermal and oxygen species. As established in that study, the polymer exhibits outstanding thermal and thermal oxidative stability, primarily due to the presence of thermally stable triazine rings and aromatic structures. These properties render the polymer highly resistant to ignition and endow it with exceptional flame retardancy.

To further prove this, an XPS investigation was performed on the char to gain a more comprehensive understanding of the surface chemistry of the composites. The high-resolution XPS spectra of the char residues (as shown in Figures S5-9) provided insights into the surface chemistry of the char and bonding characteristics of various elements. The char residue of all composite laminates primarily contains C, P, O, N, and Si. Among them, the C_1s_ spectrum peaks were centered at around 284.80 eV (C–C and C–H of the aliphatic and aromatic species), 286.04 eV (C–O group), 289.27 eV (C=O group), and 282.65 eV (Si–C group)^70^. The O_1s_ spectrum exhibited two peaks at around 531.86 eV (P=O or C=O groups) and 533.17 eV (C–O–C groups) for gFRP, and a new peak at 532.97 eV in hFRP composite laminates^70^. The N_1s_ spectrum displays two peaks at around 400.46 eV (stable C–N bond in the six-membered ring in isocyanurate species) and 398.28 eV (N–P bond) in the hybrid composites laminates char residue^71^. The P_2p_ spectrum exhibited two peaks at around 132.98 eV (P=O group) and 133.85 eV (P=N group). The hFRP composite laminates char has not exhibited any peaks for Si_2p,_ but the gFRP and hybrids composite laminates char peaks at around 102.84 eV (attributed to the silicone oxides structures)^71^. Overall, the results indicate that during the ignition of the composite laminates, the char formation is primarily attributed to the presence of thermally stable isocyanurate and phosphine oxide structures^71^. This char formation effectively delays thermal decomposition and inhibits heat transfer from the combustion zone to the substrate. Additionally, the formation of barrier-like char layers retards the propagation of combustible pyrolysis volatiles, thereby mitigating the fire risk. A similar conclusion has also been proposed by Feng et al.^38^.

The same three-point bending test was conducted on the burned sample after 40 s ignition to measure its residual strength. Since the hFRP composite laminates were burned out and collapsed in 30 s, these samples could not participate in further mechanical tests. The stress–strain curves are presented in Fig. 3d, and results are summarized in Table 3. The results indicate that the burnt gFRP and hybrid composite laminates (hFRP-G1 and hFRP-G2) have their respective residual strength up to 90.57 MPa, 50.70 MPa, and 61.50 MPa, which are 96.10%, 66.78%, and 83.53% of the corresponding strength of the virgin samples. This residual strength after burning provides continued structural integrity and safety, allowing for potential post-fire usability and retrofitting opportunities, offering cost savings and reduced downtime.

## Conclusions

In this study, a newly synthesized high-temperature shape memory polymer was employed to fabricate hybrid glass fiber and natural hemp fiber reinforced polymer composite laminates. Conforming to the normal stress distribution in laminated composite beams, the synthetic glass fibers were stacked atop a central core of natural hemp fiber. The hybrid composites exhibited a sevenfold increase in bending strength compared to pure hemp FRP composites. In addition, the hybrid composites show higher peak impact force and higher ductility index than those of the laminates made of solely glass fiber reinforcement. Moreover, the hybrid composites displayed favorable shape memory characteristics, with a shape fixity ratio of more than 52% and a shape recovery ratio of more than 71%. The composite laminate gained effective flame retardancy by incorporating TPO as both photo-initiator and flame-retardant element, validating the positive interaction between phosphine oxide structures and the isocyanurate ring. After 40 s continuous exposure to an open flame, the hybrid composite still maintains 83.53% of the bending strength of the virgin laminated composite without burning. This research demonstrates that integrating natural fibers with a small proportion of synthetic fibers can produce composites, which possess mechanical and functional properties comparable to those reinforced solely by synthetic fibers. The combination of natural fibers and a smart polymer matrix presents opportunities for lightweight structures in various applications, given the exceptional thermal stability, superior mechanical properties, and multifunctionality of the hybrid laminated composites in challenging environments.

### Supplementary Information

Supplementary Information.

## Data Availability

All data generated or analyzed during this study are included in this published article and its supplementary information file.
