# Supplementary material for: Hybrid hemp/glass fiber reinforced high-temperature shape memory photopolymer with mechanical and flame-retardant analysis
Source: Sci Rep. 2023 Oct 19;13:17830. doi: 10.1038/s41598-023-44710-6 (PMC10587156; doi:10.1038/s41598-023-44710-6)
Supplement: Supplementary file 1 — Supplementary Information. [file 41598_2023_44710_MOESM1_ESM.docx]

***­­****Supplementary Information:*

**Hybrid hemp/glass fiber reinforced high-temperature shape memory photopolymer with mechanical and flame-retardant analysis**

Sakil Mahmud^a^, John Konlan^a^, Jenny Deicaza^b^, and Guoqiang Li^a,b,*^

^a^ Department of Mechanical and Industrial Engineering, Louisiana State University, Baton Rouge, LA 70803, United States of America.

^b^ Department of Mechanical Engineering, Southern University and A&M College, Baton Rouge, LA 70813, United States of America.

*Corresponding author: G. Li (E-mail: lguoqi1@lsu.edu).

**Table S1:** Typical mechanical properties of hemp fiber and its chemical composition^1-4^

| **Properties of fibers** | | | **Chemical composition** | |
| --- | --- | --- | --- | --- |
|  | **Glass fibers** | **Hemp fibers** | **Glass fibers** | **Hemp fibers** |
| Density (g/cm^3^) | 2.62 | 1.4 | 52.4% SiO_2_ | 77.5% Cellulose |
| Tensile strength (MPa) | 3400 | 550 – 900 | 14.4% Al_2_O_3_ | 10.0% Hemicellulose |
| Elastic modulus (GPa) | 73 | 70 | 10.6% B_2_O_3_ | 6.8% Lignin |
| Specific strength (MPa/(g/cm^3^)) | 1297 | 393 – 643 | 17.2% CaO | 2.9% Pectin |
| Specific modulus (MPa/(g/cm^3^)) | 28 | 50 | 4.5% MgO | 0.9% Fat & wax |
| Elongation at failure (%) | 4.8 | 1.6 | 0.8% Others | 1.8% Water soluble materials |
| Moisture absorption (%) | N/A | 6 – 12 |  |  |
| Cost ($/kg) | 2.47 | 0.67 |  |  |


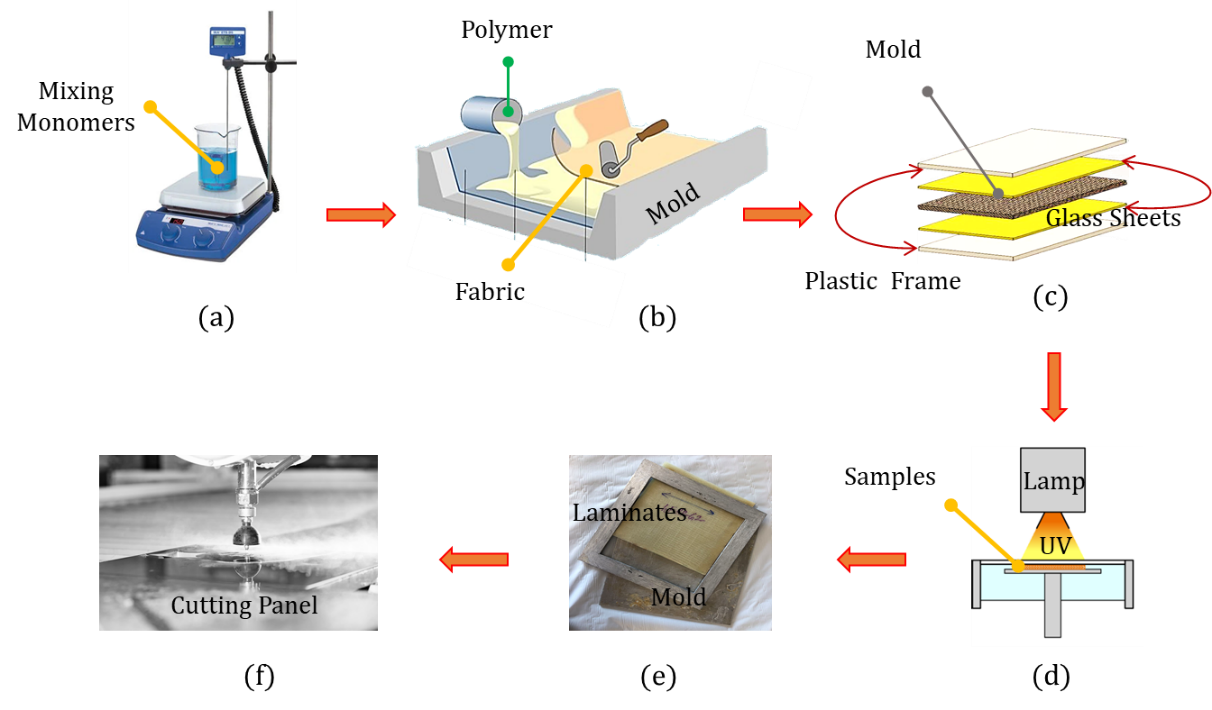


**Figure S 1**: (a) Mixing of TIA monomer and TPO photoinitiators, (b) lamination of fabrics using hand-layup, (c) laminate sandwiched in the mold with the glass sheet and open plastic frame, and the C-clamping to exert pressure, (d) UV-curing of composite laminates, (e) demolding the sandwiched laminate after UV-curing, and (f) cutting of boards into rectangular beams.


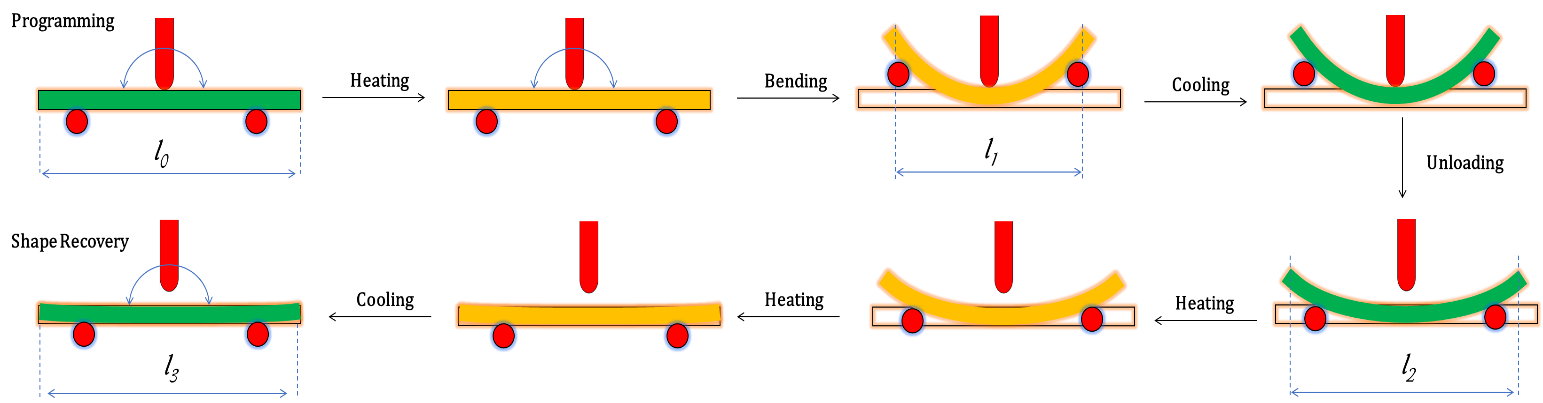


**Figure S 2**: A schematic diagram of bending programming and recovery cycle of composite laminates.


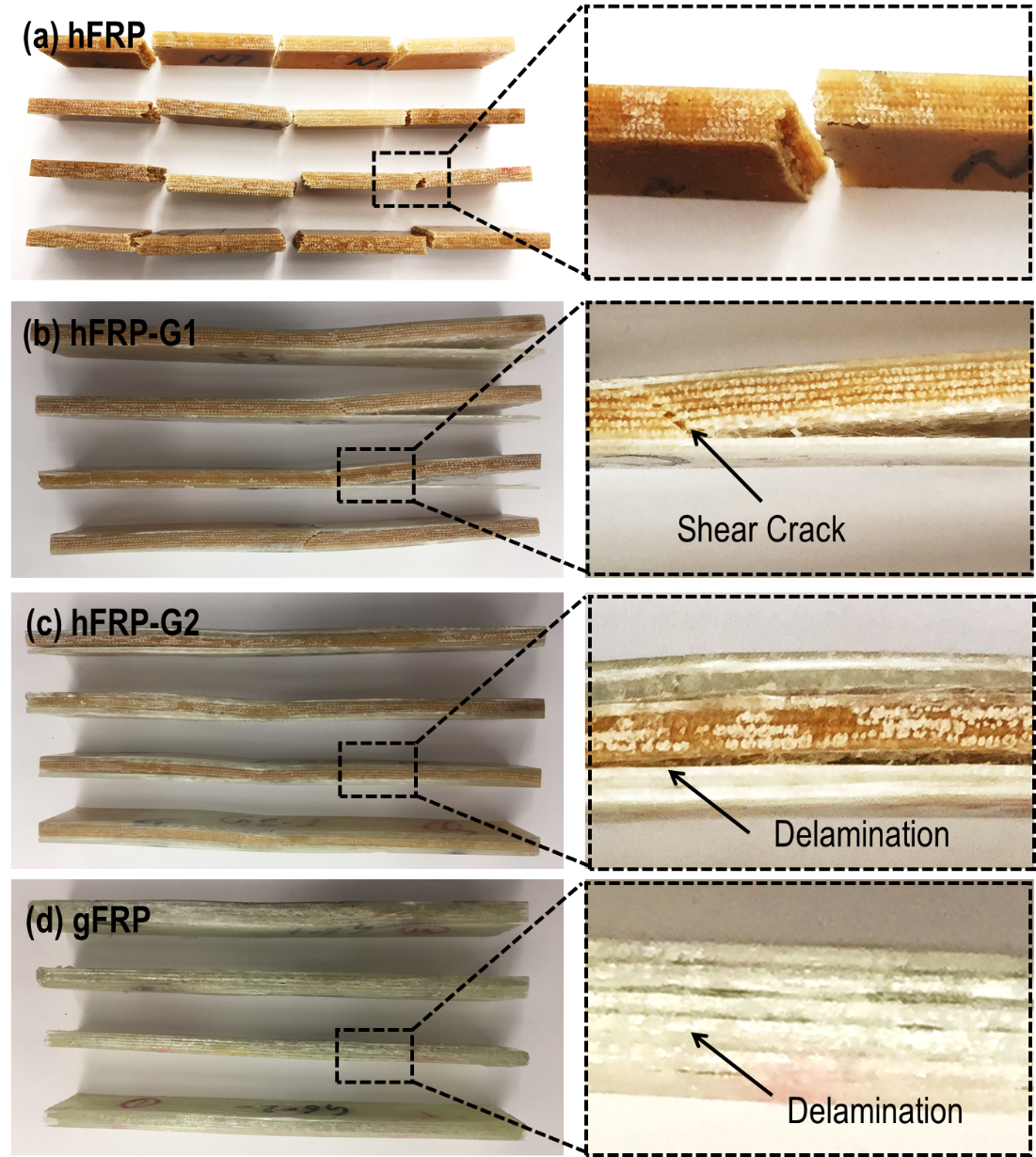


**Figure S 3:** Typical failure of laminates upon low-velocity impact: (a) hFRP, (b) hFRP-G1, (c) hFRP-G2, and (d) gFRP laminate composites.


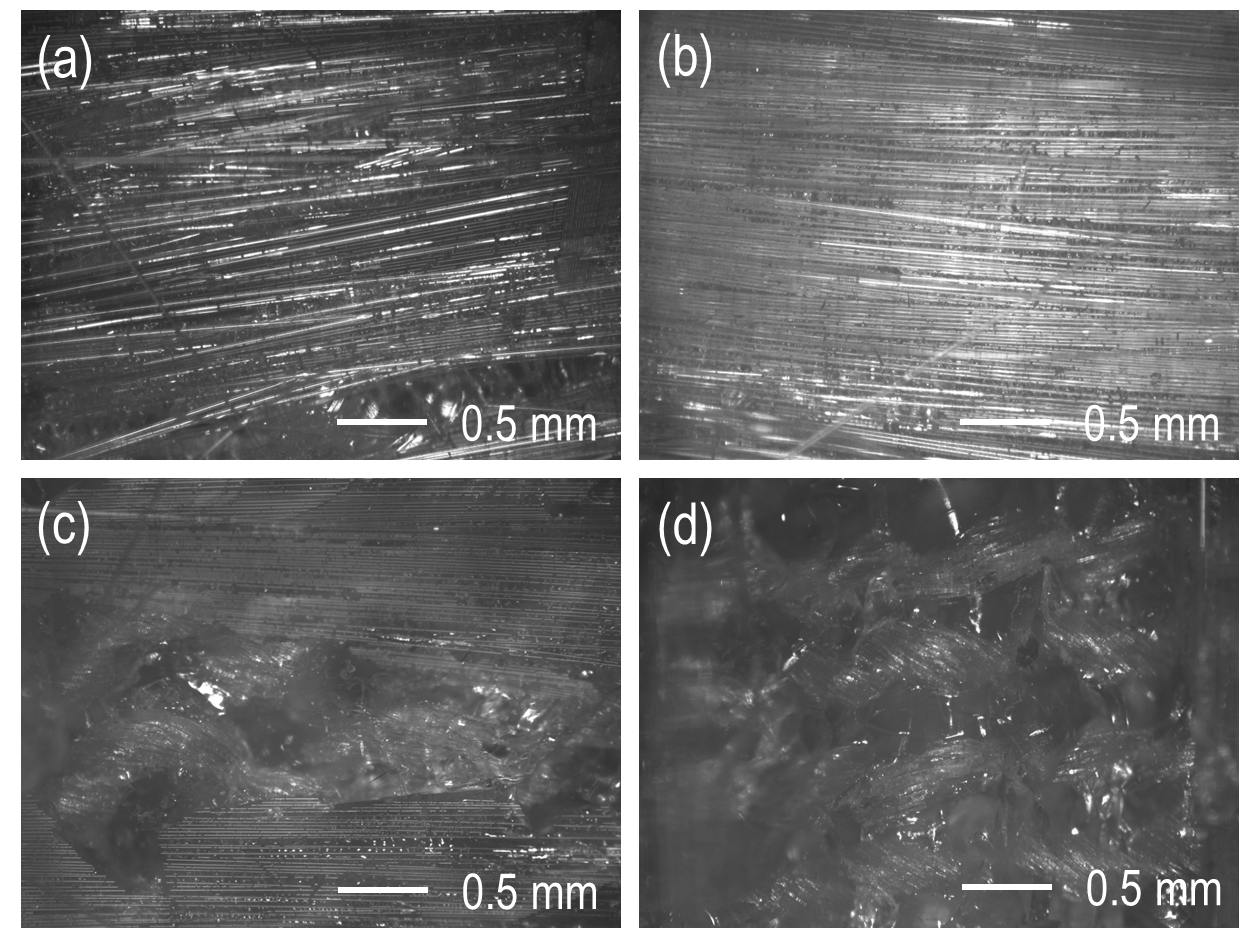


**Figure S 4:** Optical microscopic images of the delaminated surface after low-velocity impact. (a) gFRP composites laminate, (b) hybrid composites: fracture surface on the glass fiber side, (c) hybrid composites: fracture surface on the hemp fiber side, and (d) hybrid composites: cross-sectional image of the hemp fiber reinforced polymer core.


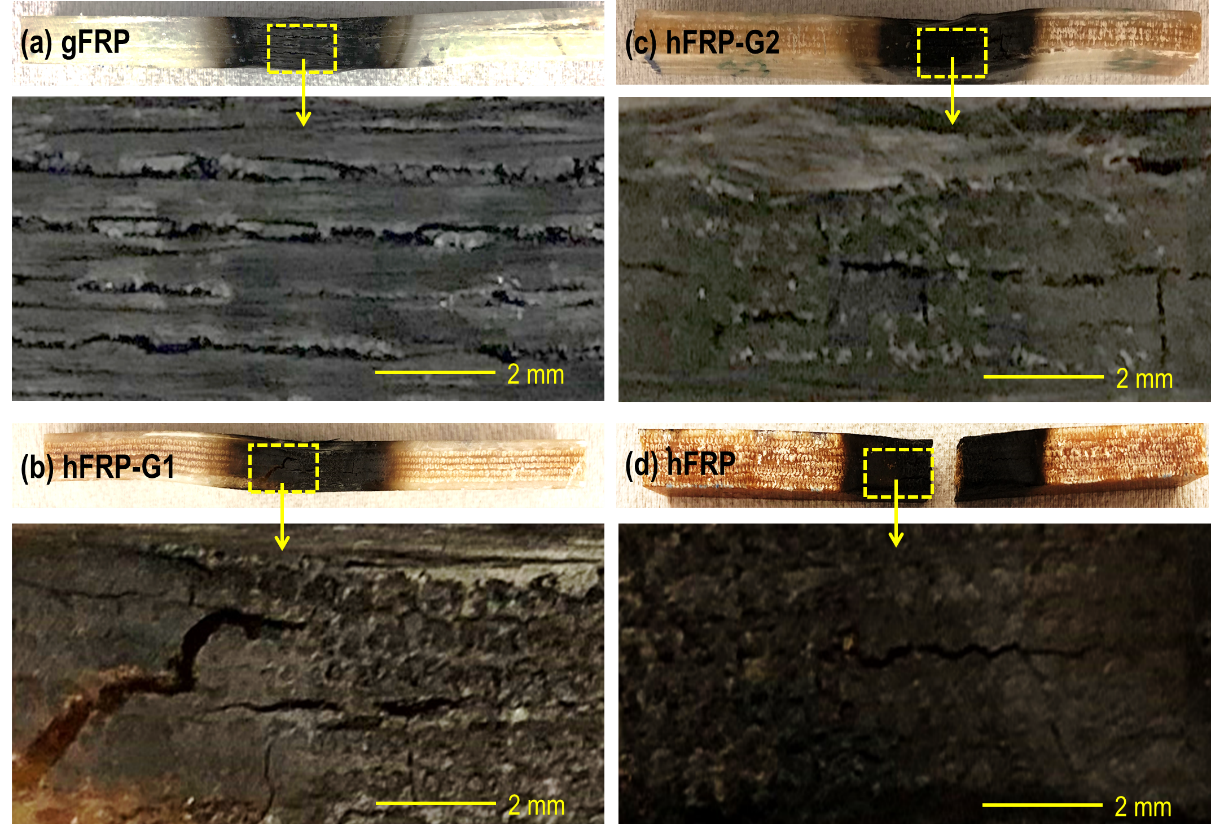


**Figure S 5:** Typical failure of laminates upon low-velocity impact after burning 40 s: (a) gFRP, (b) hFRP-G1, (c) hFRP-G2, and (d) hFRP laminate composites.


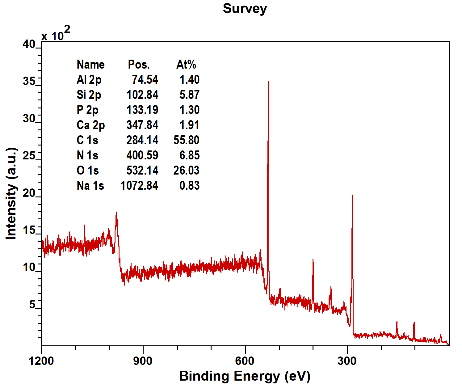

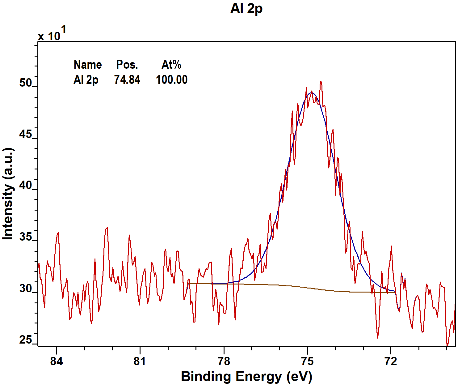

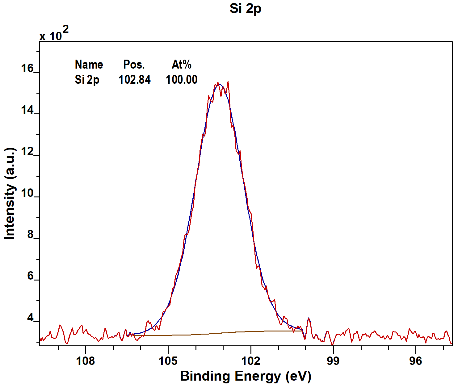


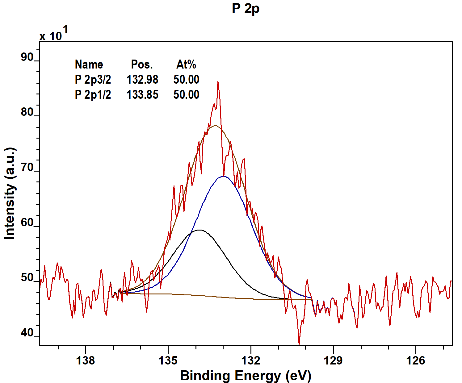

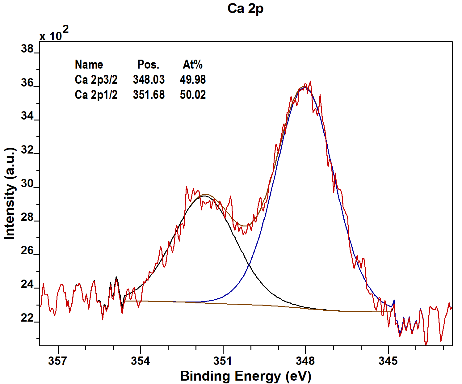

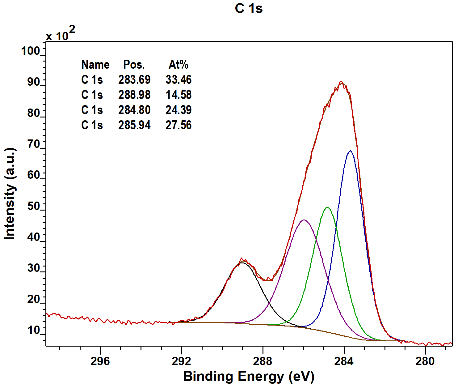


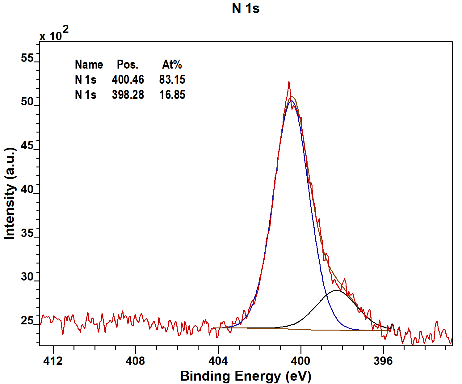

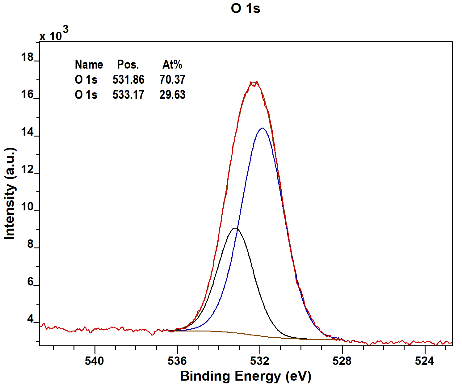

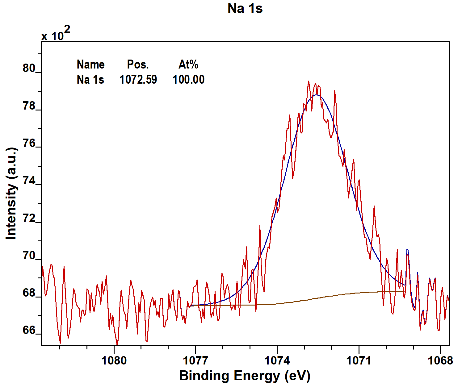


**Figure S 6:** XPS curves of gFRP laminate composites char.


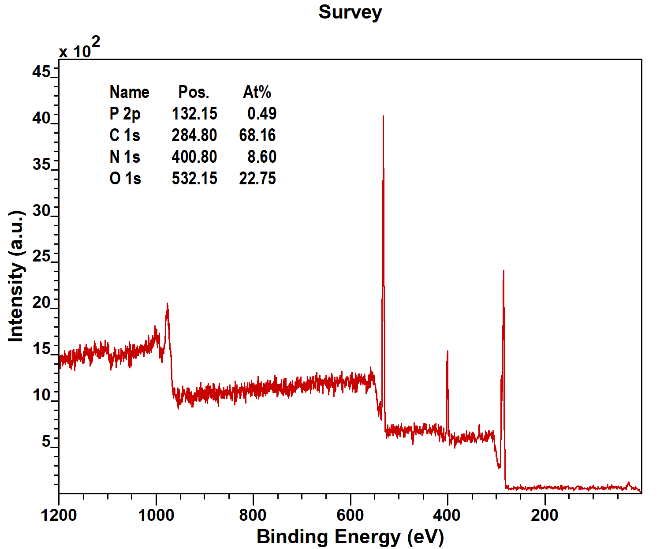


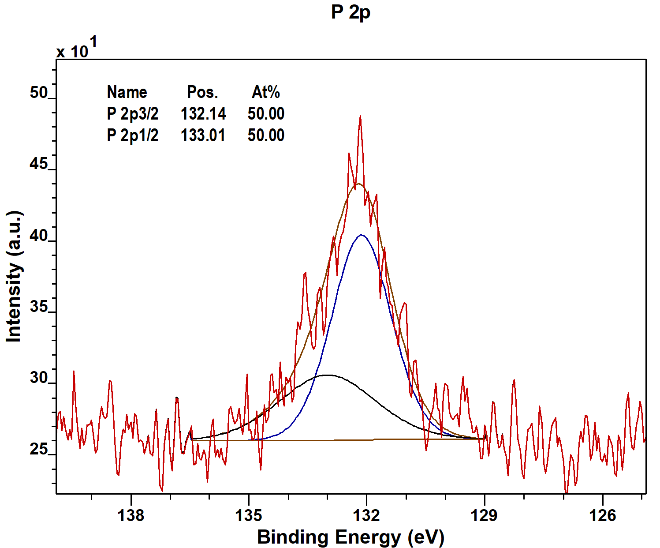

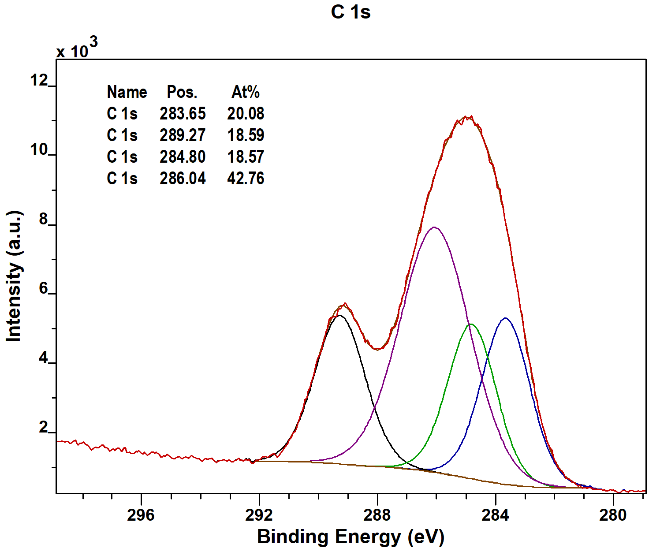


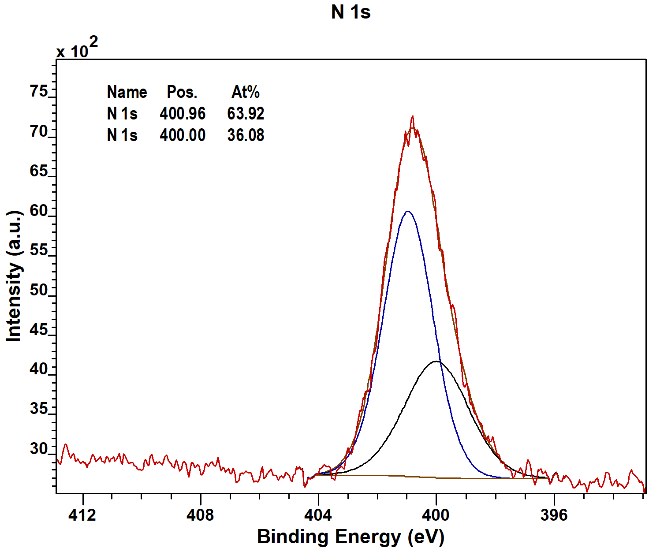

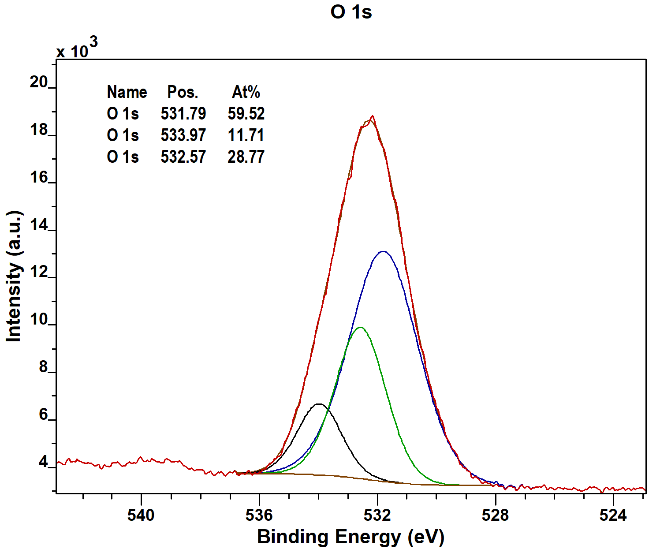


**Figure S 7:** XPS curves of hFRP laminate composites char.


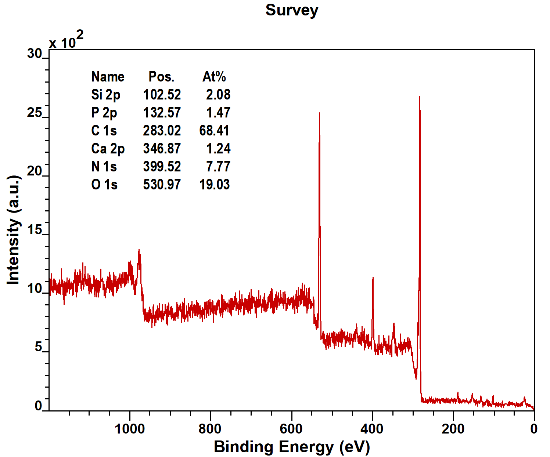


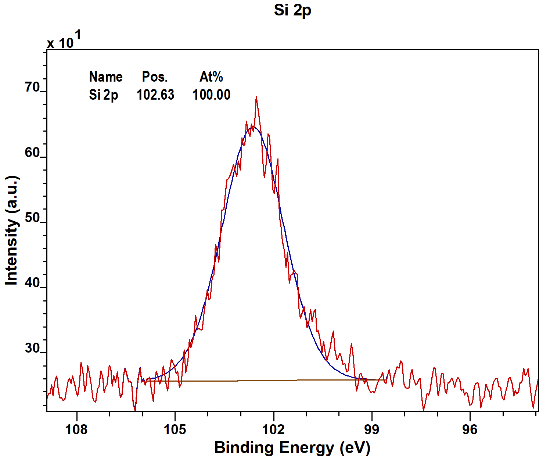

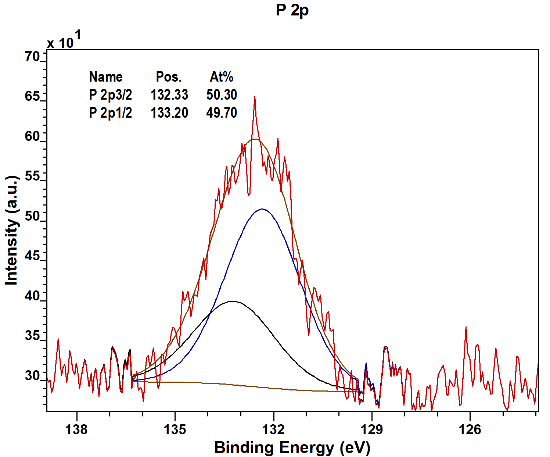


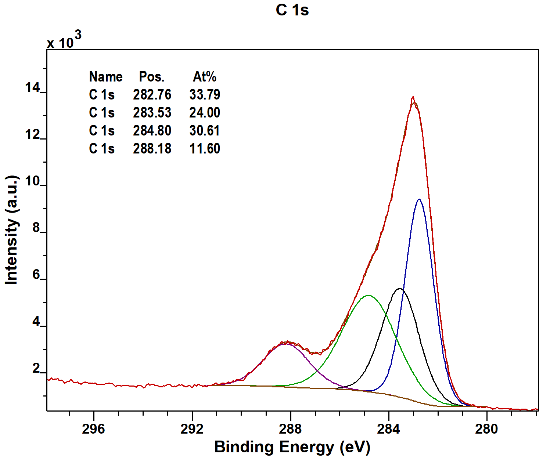

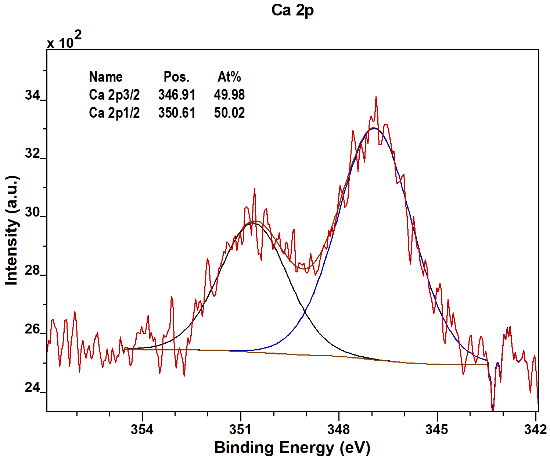


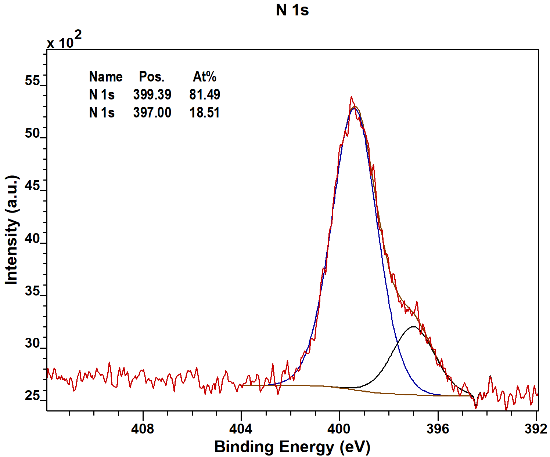

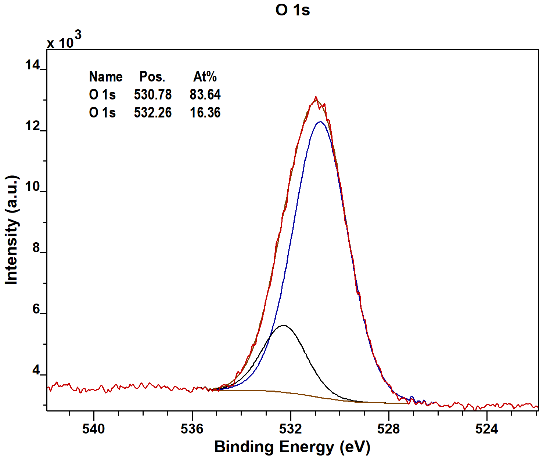


**Figure S 8:** XPS curves of hFRP-G1laminate composites char.


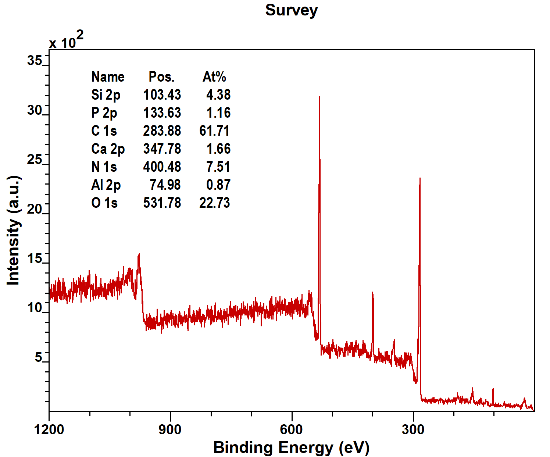

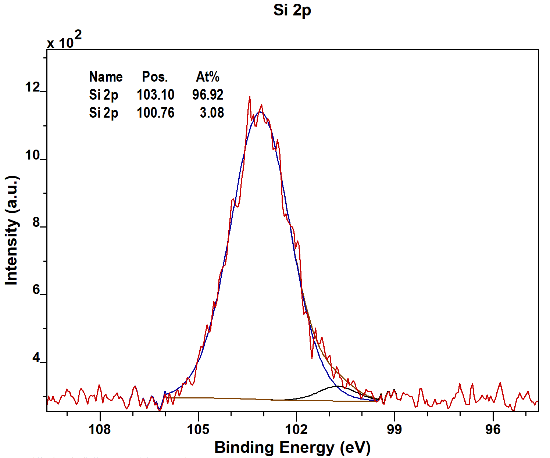


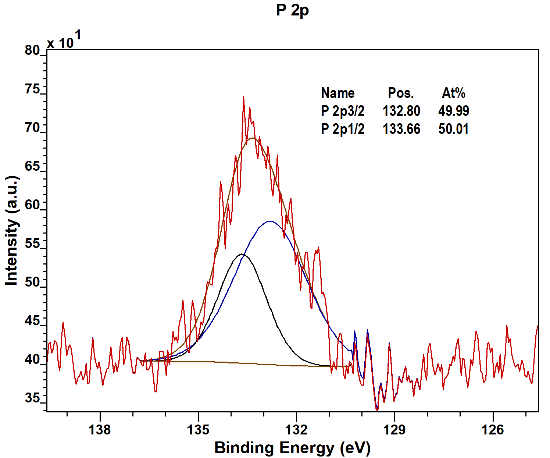

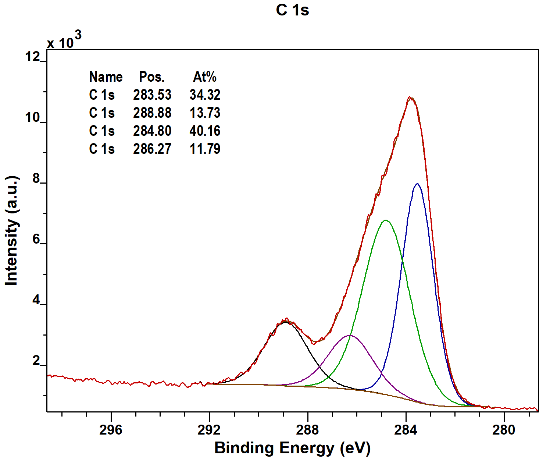


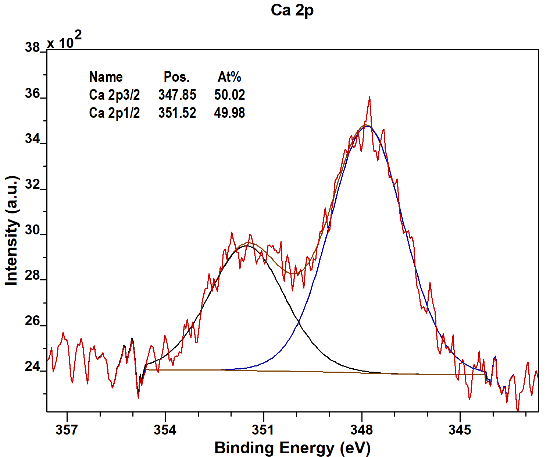

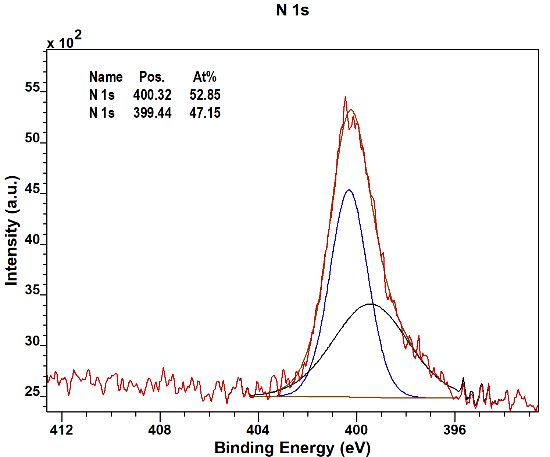


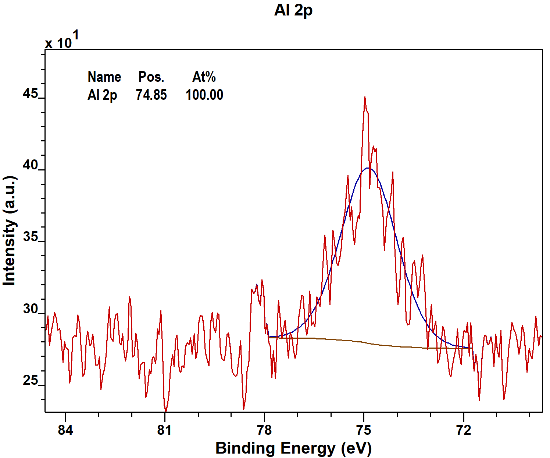

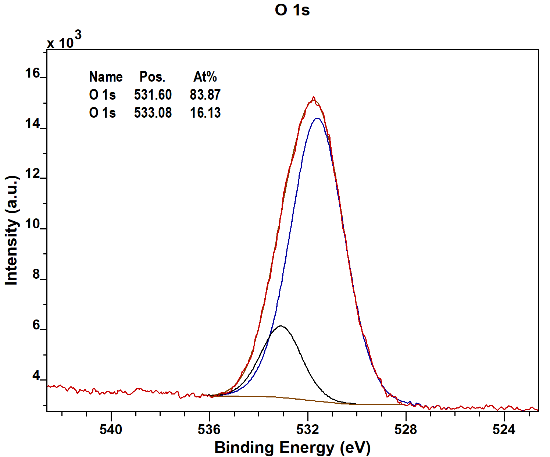


**Figure S 9:** XPS curves of hFRP-G1 laminate composites char.

**References:**

1 Shahria, S. Fabrication and property evaluation of hemp–flax fiber reinforced hybrid composite. *Cellulose* **7**, 17-23 (2019).

2 Lu, N., Swan, R. H. & Ferguson, I. Composition, structure, and mechanical properties of hemp fiber reinforced composite with recycled high-density polyethylene matrix. *Journal of Composite Materials* **46**, 1915-1924, doi:10.1177/0021998311427778 (2012).

3 Islam, M., Pickering, K. & Foreman, N. Influence of alkali treatment on the interfacial and physico-mechanical properties of industrial hemp fibre reinforced polylactic acid composites. *Composites Part A: Applied Science and Manufacturing* **41**, 596-603 (2010).

4 Bodig, J. & Jayne, B. A. Mechanics of wood and wood composites. (1982).
